# Single-Molecule Identification of the Isomers of a Lipidic Antibody Activator

**DOI:** 10.1021/acs.jpclett.4c00164

**Published:** 2024-06-27

**Authors:** Benjamin Mallada, Federico Villalobos, Beatriz Donoso, Raquel Casares, Giovanna Longhi, Jesús I. Mendieta-Moreno, Alejandro Jiménez-Martín, Ali Haïdour, Ravin Seepersaud, Lakshmi Rajagopal, Bruno de la Torre, Alba Millán, Juan M. Cuerva

**Affiliations:** ‡ Institute of Physics, Czech Academy of Sciences, 16200 Prague, Czech Republic; § Regional Centre of Advanced Technologies and Materials, Czech Advanced Technology and Research Institute (CATRIN), Palacký University Olomouc, 78371 Olomouc, Czech Republic; ∥ Departamento de Química Orgánica, Unidad de Excelencia de Química Aplicada a la Biomedicina y Medioambiente, C. U. Fuentenueva, Universidad de Granada, 18071 Granada, Spain; ⊥ Dipartimento di Medicina Molecolare e Traslazionale, Universitá di Brescia, Viale Europa 11, 25121 Brescia, Italy; # Instituto de Ciencia de Materiales de Madrid (ICMM), Consejo Superior de Investigaciones Científicas (CSIC), 28049 Madrid, Spain; ∇ Faculty of Nuclear Sciences and Physical Engineering, Czech Technical University, 11519 Prague, Czech Republic; ○ Unidad de Resonancia Magnética Nuclear, Centro de Instrumentación Científica, Universidad de Granada, Paseo Juan Osorio s/n, 18071 Granada, Spain; ◆ Center for Global Infectious Disease Research, Seattle Children’s Research Institute, Seattle, Washington 98109, United States; ¶ Department of Global Health, University of Washington, Seattle, Washington 98105, United States; ▲ Department of Pediatrics, University of Washington, Seattle, Washington 98105, United States

## Abstract

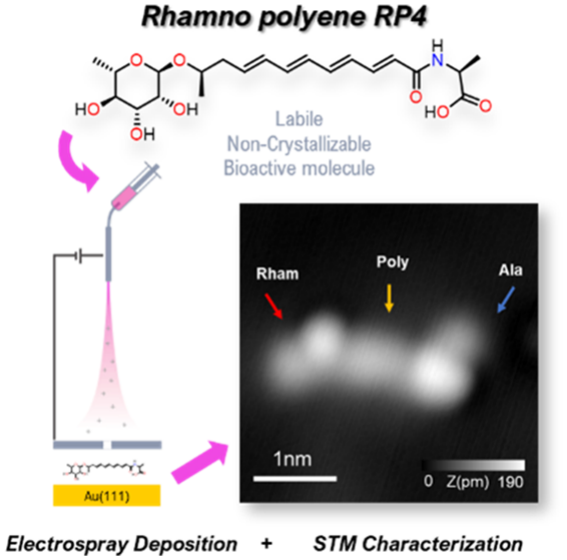

Molecular structural elucidation can be accomplished by different techniques, such as nuclear magnetic resonance or X-ray diffraction. However, the former does not give information about the three-dimensional atomic arrangement, and the latter needs crystallizable solid samples. An alternative is direct, real-space visualization of the molecules by cryogenic scanning tunneling microscopy (STM). This technique is usually limited to thermally robust molecules because an annealing step is required for sample deposition. A landmark development has been the coupling of STM with electrospray deposition (ESD), which smooths the process and widens the scope of the visualization technique. In this work, we present the on-surface characterization of air-, light-, and temperature-sensitive rhamnopolyene with relevance in molecular biology. Supported by theoretical calculations, we characterize two isomers of this flexible molecule, confirming the potential of the technique to inspect labile, non-crystallizable compounds.

One of the most relevant advances in human knowledge has been the understanding of biological processes and its dramatic consequences for human health.^1,2^ Such processes usually rely on a fine interplay between organic-based entities, which, in many cases, are labile outside the biological protecting media. The molecular interactions are highly dependent upon functionality and also geometry at the molecular level. Therefore, determining the structure of such organic molecules is critical to rationalizing their mechanism of action. Routinely, nuclear magnetic resonance (NMR) techniques are used to assign the structure of organic compounds. However, these techniques are limited in their capability to discern the constitution and configuration of molecules, which lead on occasions to wrong assignments.^3,4^ Furthermore, the outputs come from a myriad of individual molecules, which introduces a notable drawback, as the precise assignment or manipulation of individual structures lies beyond the scope of this methodology. A more precise approach to structural analysis relies on X-ray diffraction of single crystals. In such a case, the distance and relative positions between the atoms are acquired, showing the connectivity (molecular constitution) and three-dimensional (3D) structure (molecular configuration). Despite its undoubtable utility, a key limitation remains because it is not always possible to grow high-quality single crystals.^5^ Although crystalline sponges (CSs)^6^ have been proposed as an alternative, the technique requires a careful selection of the CS, which varies depending upon the target molecule.^7^ Moreover, this technique continues to analyze an ensemble of molecules without any possible molecular manipulation. Cryogenic electron microscopy (cryo-EM) has emerged as another tool allowing for the characterization of macromolecules.^8^ Despite the impressive results that can be achieved, atomic manipulation cannot be performed on the samples. Therefore, the development of an alternative technique of general use is of utmost demand for the structural identification and manipulation of organic compounds.

Technological advances have allowed for the imaging of molecules with unprecedented resolution on different surfaces, thus creating a potent tool for electronic and structural analysis.^9−11^ In fact, cryogenic scanning tunneling microscopy (STM) allows for real-space imaging of individual molecules deposited on a surface under ultrahigh vacuum (UHV) conditions with sub-nanometer resolution, resulting in a unique instrument for the study of molecular structures, molecular interactions, and reactivity in a controlled environment.^12,13^ Remarkably, tip manipulation also makes a direct non-destructive interaction with the substrate possible at the unimolecular level. That characteristic allows for stability or integrity studies among others.^14^ Nevertheless, some intrinsic drawbacks related to sample deposition have limited the studies mainly to molecules with high thermal stability as a result of the UHV sublimation process required for sample deposition. Considering the vast number of potential organic structures, innovations capable of dealing with thermally labile or even unstable molecules would be in the forefront of the technique.^15−17^ One potential solution is the combination of STM with electrospray deposition (ESD) or electrospray ion beam deposition (ES-IBD),^18,19^ which has been demonstrated for the imaging of numerous organic molecules.^20−30^ The soft landing of molecules on the atomically clean surface is an excellent alternative for transferring them as a result of its compatibility with many typical organic functional groups. Therefore, the ESD technique presents unique characteristics allowing for the visualization and manipulation of highly sensitive molecular architectures, including those out of the scope of X-ray diffraction techniques, at the unimolecular level in a controlled environment. However, the characteristic exponential decay of the tunneling current with the tip–sample distance still prevents its use for large nonplanar molecular structures where it is more conveniently imaged and characterized using other techniques, such as cryo-EM (e.g., β-galactosidase).^31^

Although some examples have been reported,^28,32,33^ the study of synthetic compounds with relevant biological interest has been less explored and would open new opportunities for this technique. Therefore, we focus on a synthetic lipidic molecule (RP4 in Figure 1a), which mimics a more complex natural product: granadaene.^34,35^ This latter longer polyene is cytotoxic/hemolytic and is a key virulence factor promoting all facets of group B *Streptococcus* (GBS) disease.^36−42^ Although GBS typically resides in the lower genital tract of healthy women, perinatal GBS infections lead to preterm births, stillbirths, or severe diseases in newborns.^43−45^ Currently, no vaccine exists to prevent GBS infections in humans. This is due in part to the difficulty in neutralizing these toxic lipids and challenges in generating non-toxic antigenic lipids. Despite these challenges, we recently showed that RP4 is a non-hemolytic lipid, and RP4 immunization conferred the production of antibodies that inhibited granadaene-mediated hemolysis and diminished GBS infection in both non-pregnant and pregnant mice.^46,47^ RP4 is composed of three distinct components, namely, a rhamnose unit, a terminal amino acid group (alanine), and a main chain consisting of four conjugated double bonds (the acronym RP4 refers to a rhamnopolyene with four double bonds). RP4 shows ideal characteristics for this STM–ESD proof-of-concept study: (i) the structure is known, which is relevant for the validation of the results; (ii) it is polyenic in nature, with RP4 being sensitive to air, temperature, and light and, therefore, labile and ambient unstable; and (iii) it is a non-crystallizable liquid. Moreover, it represents a model for a family of biologically relevant polyenes whose structure is not completely understood, being difficult to manage as a result of its instability. The RP4 determination by this technique paves the way for future structural assignment and understanding of pathogenic behavior of human-threatening GBS bacteria.

**Figure 1:**
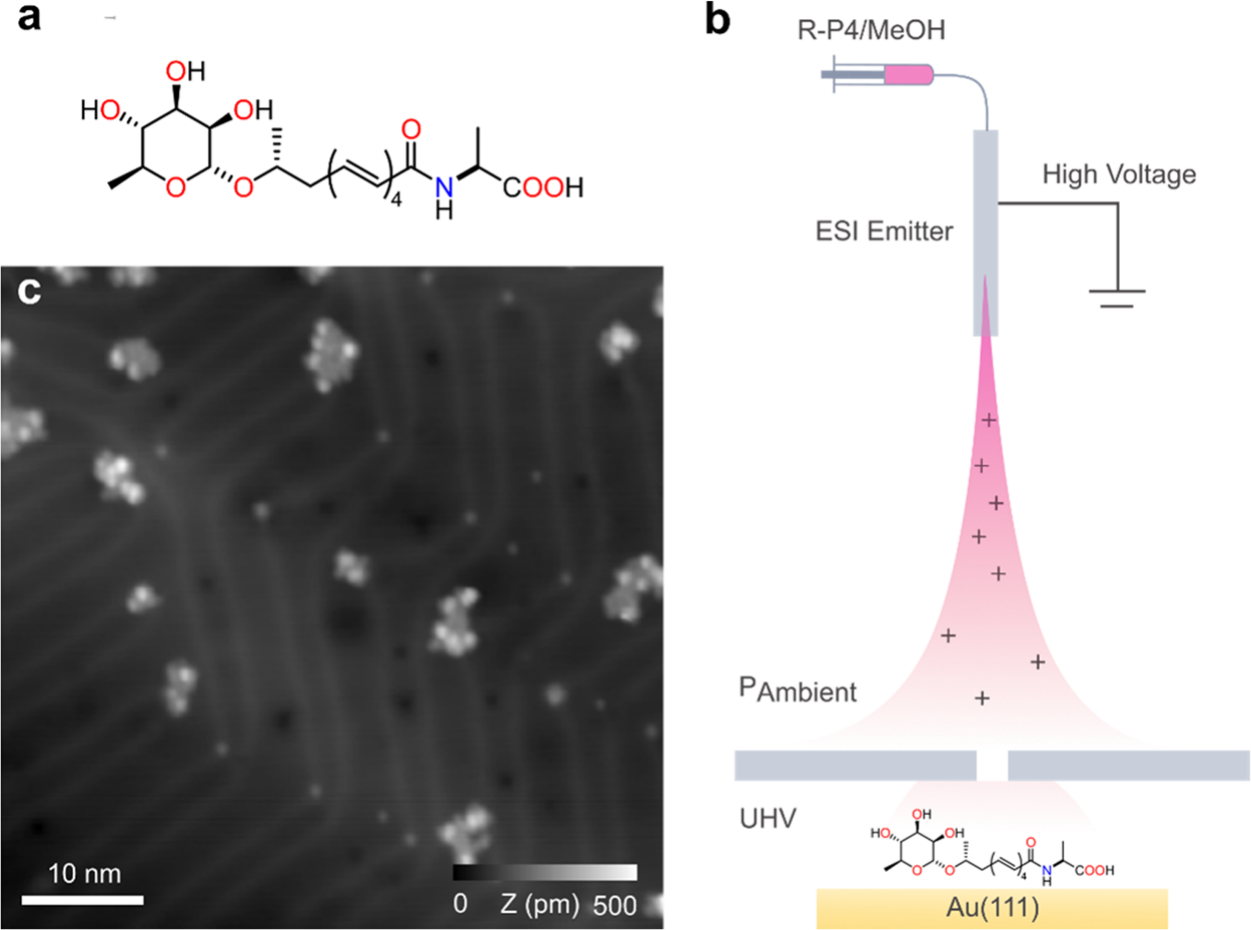
Electrospray deposition on Au(111) of RP4. (a) Chemical structure of RP4 with the rhamnose group (left), the polyenic chain, and the alanine group (right). (b) Schematic of the ESD from the liquid solution to the Au(111) sample in UHV. (c) Constant-current STM overview of Au(111) after ESD of solution RP4/methanol.

Furthermore, the understanding of its structure at the unimolecular level would be a step forward in the understanding of its mode of action as a potential vaccine. Despite the significant difference between the metal surface in UHV and the liquid environment of a vaccine, this exploratory study exemplifies how direct imaging and manipulation of the RP4 molecule by STM allows us to unambiguously discriminate the configuration and structure of individual isomers on the surface.

We first prepared RP4 (Figure 1a) following a stereoselective synthetic approach, affording the all-*E* isomer as the major product (see the Supporting Information for further details). The molecular deposition of RP4 on atomically defined Au(111) was carried out under UHV conditions at room temperature, as illustrated in Figure 1b. The ESD technique was employed to deposit a solution of RP4 in methanol (2 mg/mL) onto the surface, as described in the “Experimental and Computational Methods” section. It is important to note that our setup did not include mass or energy selection. The sample was then quickly transferred to a cryogenic scanning tunneling microscope operating at 4.8 K for further examination. The large-scale STM topographs (Figure 1c) revealed that RP4 molecules tend to agglomerate into small and disordered two-dimensional clusters with typical apparent heights ranging from 260 to 500 pm (see the Supporting Information). We postulate that the aggregation of RP4 occurs at the Au surface owing to low surface diffusion barriers and large molecular mobility at room temperature, with the aggregates being stabilized by dispersion forces and hydrogen bonding involving OH of the rhamnose moiety.^28,48^ It is important to note that, despite ESD being an ionization process, it is plausible to assume that the ions neutralize rapidly upon contact with the metallic surface as a result of charge transfer with the surface.

To investigate the chemical composition of the clusters, we employed tip-induced manipulations to isolate individual molecular units for a thorough examination. The procedure involved a concise mechanical interaction between the tip and the molecule, resulting in the tip exerting a pulling force on certain parts of the cluster in the direction of fast scanning. Specifically, we deliberately modified the imaging conditions, particularly the tip–sample distance, to separate the molecular aggregates into individual units, as shown in Figure 2 (further details in the “Experimental and Computational Methods” section). Figure 2a illustrates the initial state before cluster manipulation. When the bias voltage was ramped down (from 501 to 1 mV) while scanning the cluster, the tip–sample height was lowered, thus increasing the tip–cluster interaction, as shown in Figure 2b. This step was critical to achieve the separation of the molecular aggregates into individual units. The outcome of cluster manipulation is depicted in Figure 2c, where a single object can be clearly identified. Crucially, this entity possesses a quantifiable length of 2.7 nm, akin to the theoretical extension of the RP4 molecule in the STM-calculated images. This procedure enabled us to discern the chemical content of the clusters and provided a more comprehensive understanding of the properties and behavior of RP4 on the surface. The procedure was repeated over several aggregates, systematically leading to the isolation of single objects with similar characteristics on the surface. However, this method of isolation does not necessarily preclude the presence of other chemical entities on the surface despite only observing single molecules during the cleaning process. Remarkably, following the manipulation of the cluster, no residual material was observed in the vicinity of the scanned region nor were there any alterations to the tip apex. As a consequence, the complete identification and assignment of structures to single molecules require a careful assessment of the surface chemistry of the clusters.

**Figure 2:**
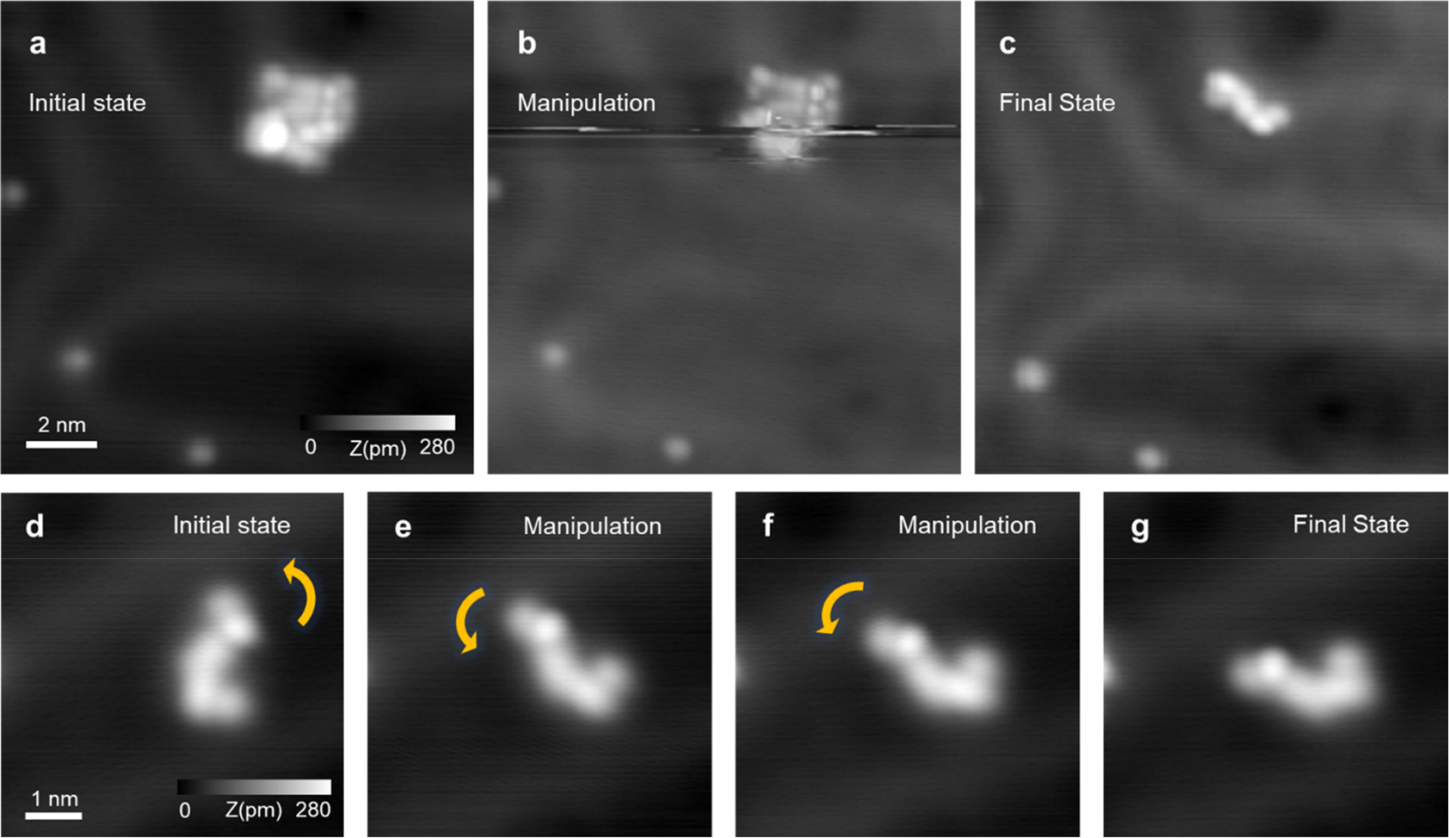
Isolation and manipulation of the RP4 isomers from clusters. (a–c) Detail in constant current STM of the separation of RP4 clusters by tip manipulation. (d–g) Sequence of STM images displaying a tip-induced manipulation on a single monomer from an initial state (d) to a final state (g) through a sequence of rotations (see the Supporting Information for further examples). Scanning parameters: (a) *V*_s_ = 501 mV and *I* = 10 pA, (b) *V*_s_ = 1 mV and *I* = 10 pA, (c) *V*_s_ = 51 mV and *I* = 10 pA, and (d–g) *V*_s_ = 501 mV and *I* = 10 pA.

Next, we conducted controlled molecular manipulations on the single objects using STM to further demonstrate the chemical integrity and stability of the single RP4 molecule. Specifically, we induced rotations and translations of individual RP4 molecules using the STM tip, as illustrated in panels d–g of Figure 2, following isolation of the molecule, as described above. This process was executed sequentially, with each step being imaged to track the position of the molecule from an initial state to a final state. The full manipulation sequence provided compelling evidence that the characteristic functional groups of RP4 remained intact throughout the manipulation process and that the molecule was covalently bonded, with no evidence of tip-induced isomerizations or irreversible changes. These results further reinforce the chemical and stereochemical stability of the RP4 molecule and its potential applicability.

We now shift our focus to examining the stereochemistry of RP4 molecules on the surface. This molecule is relatively complex with a huge number of potential conformations in solution. Full understanding the conformational space of RP4 is crucial to comprehend its potential interactions with other biomolecules and its relevance to biomedical applications. Moreover, the main conformer present in solution may not always be involved in molecular recognition or docking processes.

Two different isomers, RP4-1 and RP4-2, were experimentally observed, with the first isomer being much more commonly found (Figure 3). In both RP4 isomer images, we can identify the central polyenic chain flanked by two bright protrusions that correspond to rhamnose and the terminal amino acid. The main difference lies in the more curved central part of RP4-2 (Figure 3f). The structural assignment of such isomers was carried out with the aid of theoretical calculations. A completely unbiased conformational search *in vacuo* with the CREST tool^49^ gives a prevalence of folded structures in which the sugar and amino acid terminus are hydrogen-bonded, yielding a curved polyene chain not corresponding to the observed shape (see section 5.1 of the Supporting Information). These curved structures are not likely to be energetically favored on the metallic surface because favorable alkene–surface interactions are avoided. Therefore, it is not surprising that the gold surface was limiting the number of 3D arrangements compared to the vacuum. It is also worth noting that such arrangements may not be more frequent in solution. In that way, we can analyze conformations that are statistically hidden in solution. Considering that molecules arrive to the surface from solution/vacuum, the initially observed aggregates (Figure 2a) could be related with very different RP4 conformers. Nevertheless, manipulations performed to isolate the single molecule drive the system toward RP4 flat isomers to maximize the surface−π interactions. To find conformers suitable for testing on a Au surface by the calculations described in the following, a new conformational search for the all *E* isomer was carried out maintaining the *trans*-planar structural characteristic fixed while exploring all conformational possibilities for the two moieties: the rhamnose part and the amino acid (see the “Experimental and Computational Methods” section for general details and the Supporting Information for the preliminary study of the two independent parts). Two principal conformers were then identified that are partially folded (Table S3 of the Supporting Information), presenting rhamnose parallel to the polyene chain. Although they are challenging for STM elucidation, simple rotations around the rhamnose polyene bridge give a realistic conformation on the surface (see Figure S10 of the Supporting Information for a comparison of the folded geometry versus the extended geometry). This folding is energetically favorable via an energy minimization from the interactions of oxygenated functionalities with the metallic surface. We performed computational simulations with the quantum mechanics (QM)/molecular mechanics (MM) approach^50^ to characterize the geometry of the RP4 molecule on the surface for both isomers (panels d and h of Figure 3), and the corresponding STM theoretical images were simulated with a reasonable match to the experimental images (Figure 3c versus Figure 3b and Figure 3g versus Figure 3f) (see the “Experimental and Computational Methods” section for details). Our STM-simulated images accurately replicate the visual representation of the elongated central polyenic chain and the protrusions arising from the out-of-the-surface plane configuration of both rhamnose and the terminal amino acid. Guided by theoretical conformational analysis and supported by simulated images, we elucidate that the major isomer RP4-1 corresponds to the all s-*trans* conformation (Figure 3a), whereas the minor isomer RP4-2 comes from the energetically unfavorable conformation, in which diene closer to rhamnose has a s-*cis* conformation instead of the energetically favored s-*trans* conformation found in isomer RP4-1 (Figure 3e). We cannot discard the fact that the rotation of the single bond that gives s-*cis* diene occurs during the deposition/isolation experiments as a result of the low activation barrier to access such a conformation. Remarkably, as shown in Figure 2, tip-induced manipulations of the molecule did not promote a conformational change, maintaining the molecule unaltered.

**Figure 3:**
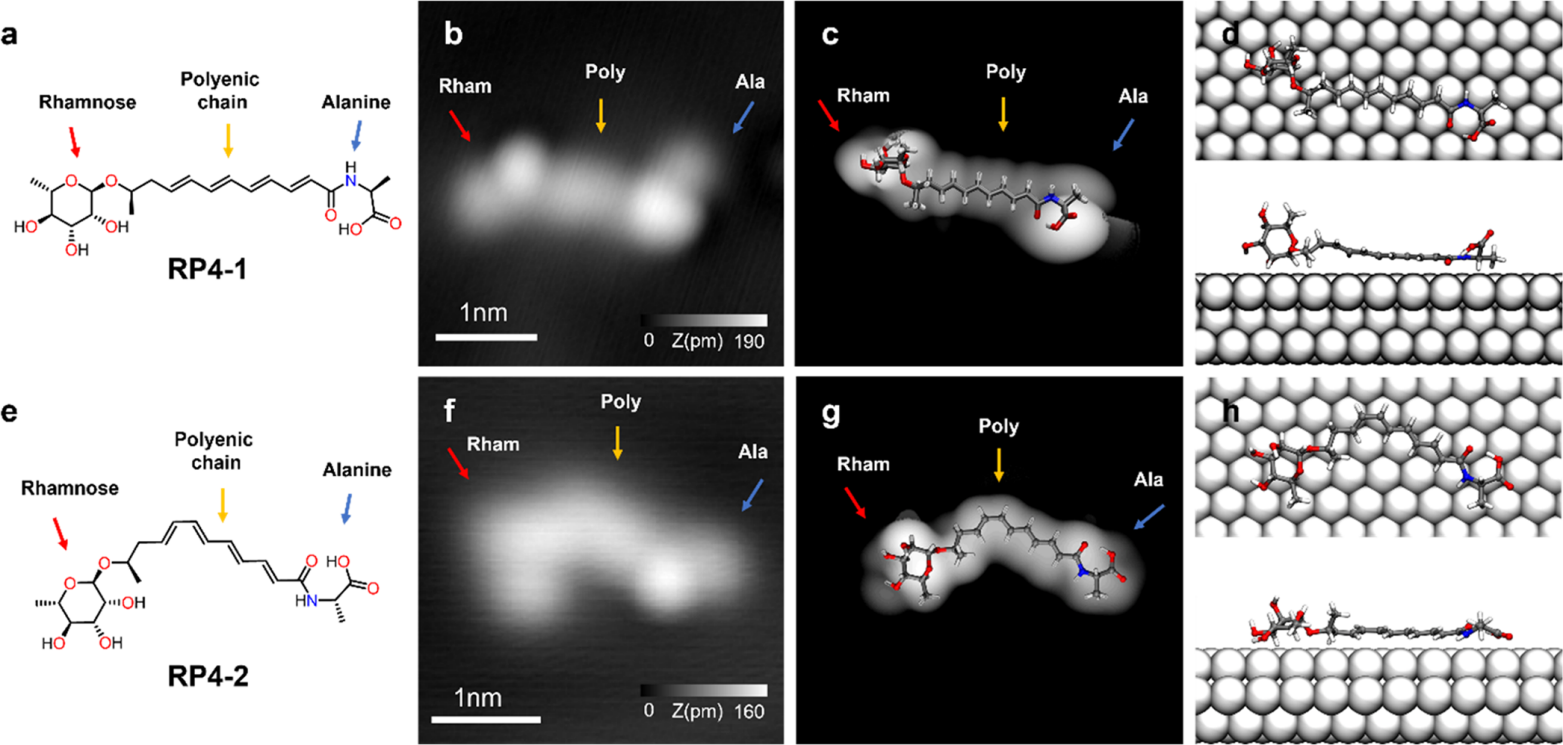
Visualization of RP4 isomers. (a and e) Chemical structures of the visualized RP4 isomers. (b and f) Corresponding constant-current STM images. The RP4-1 isomer displays the rhamnose group in one end, four *E*-alkenes in s-*trans* conformation, and the alanine amino acid in the other end. The RP4-2 isomer presents one of the diene moieties in a s-*cis* conformation. (c and g) STM-simulated images of both RP4 isomers with a superimposed model. (d and h) DFT-calculated conformations for RP4 isomers. Scanning parameters: (b) *V*_s_ = 51 mV and *I* = 10 pA and (f) *V*_s_ = 501 mV and *I* = 10 pA.

In conclusion, the present study highlights the robust potential in the synergistic application of ESD in conjunction with UHV-STM for probing the intricate understanding of labile and/or non-crystallizable molecular structures on atomically defined surfaces, achieving submolecular resolution. Specifically, we aimed to investigate the chemical structure of the rhamnopolyene lipid RP4 on Au(111). The STM images obtained from our experiments revealed the coexistence of two distinct conformational isomers of RP4 on the surface, which we further confirmed through single-molecule manipulation experiments, thereby establishing the chemical stability of the molecule. Furthermore, we performed density functional theory (DFT) calculations considering flat polyene structures to corroborate our experimental results and used such structures to simulate STM images. Our findings provide novel insights into the chemical structure of a labile complex molecule of biological interest and pave the way for future detailed investigations in this area. Further studies addressing the structural elucidation of more complex polyenes, such as granadaene, or exploring the interaction of RP4 with subsequently electrospray-deposited amino acids or carbohydrates can be envisioned.

## Experimental and Computational Methods

*Synthesis*. Unless otherwise stated, all reagents and solvents were purchased from commercial sources and used without further purification. Anhydrous tetrahydrofuran (THF) was freshly distilled over Na/benzophenone. Flash column chromatography was carried out using silica gel 60 (40–63 μm) as the stationary phase. Analytical thin-layer chromatography (TLC) was performed on aluminum sheets coated with silica gel with fluorescent indicator UV254, observed under UV light (254 nm), and stained with phosphomolybdic acid (5% methanol solution). All ^1^H and ^13^C nuclear magnetic resonance (NMR) spectra were recorded on Bruker Avance Neo (400 or 500 MHz) spectrometers at a constant temperature of 298 K. Chemical shifts are reported in parts per million (ppm) and referenced to residual solvent: CHCl_3_ (7.27 and 77.0 ppm for ^1^H and ^13^C, respectively), and MeOH (3.31 and 49.0 ppm for ^1^H and ^13^C, respectively). Coupling constants (*J*) are reported in hertz. Multiplicities are abbreviated as follows: s, singlet; br s, broad singlet; d, doublet; t, triplet; m, multiplet; dd, doublet of doublets; td, triplet of doublets; ddd, doublet of doublet of doublets; and dt, doublet of triplets. Proton assignment was carried out by two-dimensional (2D) NMR experiments: correlation spectroscopy (COSY), heteronuclear single-quantum correlation (HSQC), and heteronuclear multiple-bond correlation (HMBC), where possible. Assignment of the ^13^C NMR multiplicities was accomplished by distortionless enhancement by polarization transfer (DEPT) techniques. Electrospray ionization time-of-flight (ESI-TOF) mass spectra were recorded in a Waters Xevo G2-XS QTof. RP4 was prepared according to the literature,^46^ with some modifications detailed in the Supporting Information.

*Scanning Probe Microscopy*. The experiments were conducted within an UHV environment, where the base pressure was maintained below 5 × 10^–10^ mbar. The setup included a low-temperature scanning tunneling microscope (Createc GmbH) operating at 4.2 K. Imaging was performed using a Pt/Ir tip with the bias voltage applied to the sample. Metallic tips were achieved through controlled indentations on the exposed surface. Au(111) was prepared via standard cycles of Ar^+^ sputtering and annealing. The ESDs were carried out using a commercial system (MolecularSpray, Ltd.) outfitted with several pumping stages. Our ESD setup was run in positive mode; that is, a positive bias is applied to the emitter. The setup was linked to the UHV preparation chamber. RP4 was dissolved in methanol to form a solution with a concentration of 2 mg/mL. The deposition was performed on the sample at ambient temperature. During the spray deposition, the pressure in the chamber was less than 1 × 10^–7^ mbar. Typically, the voltages applied to the capillary ranged between 2 and 2.3 kV with necessary adjustments to maintain spray stability while keeping an approximate pumping rate of 60 μL/h for 60 min. After deposition, the sample was immediately transferred to the analysis chamber in UHV and cooled down to 4.2 K. All data were subject to standard processes using the WSxM software^51^ without any filtering or smoothing. Approximately, more than 20 RP4 molecules were considered in dozens of STM overviews of 50 × 50 nm^2^. The molecular manipulation to separate RP4 from the conglomerated islands was performed by scanning in constant current mode a conglomerate with a bias voltage of 1 mV and a tunneling current of 10 pA. After every manipulation event, the frame was rescanned in constant current mode with a bias voltage of 500 mV and a tunneling current of 10 pA. Single-molecule manipulation has been performed in constant current mode (10 pA) by ranging the bias to 1 mV.

*Theoretical Calculations*. A completely unbiased conformational search *in vacuo* was carried out with the CREST tool.^49^ After the CREST conformational search, structures have been optimized at the M06/tzvp level with the Gaussian 16 package.^52^ RP4 structures on Au(111) have been calculated using a QM/MM approach^50^ with the molecules described with Fireball DFT^53^ and the surface described with the interface force field.^54^ More details are given in the Supporting Information.

## Supporting Information Available

General details, synthetic procedures, copy of ^1^H NMR and high-resolution mass spectra, additional figures of the on-surface characterization of RP4, and computational details (PDF)

## References

[ref1] Dugas H. Bioorganic Chemistry. A Chemical Approach to Enzyme Action; Springer-Verlag: New York, 1996; 10.1007/978-1-4612-2426-6.

[ref2] Van Vranken D.; Weiss G. Introduction to Bioorganic Chemistry and Chemical Biology; Garland Science: New York, 2012; 10.1201/9780203381090.

[ref3] Nicolaou K. C.; Snyder S. A. Chasing Molecules That Were Never There: Misassigned Natural Products and the Role of Chemical Synthesis in Modern Structure Elucidation. Angew. Chem., Int. Ed. 2005, 44, 1012–1044. 10.1002/anie.200460864. 15688428

[ref4] Menna M.; Imperatore C.; Mangoni A.; Della Sala G.; Taglialatela-Scafati O. Challenges in the Configuration Assignment of Natural Products. A Case-Selective Perspective. Nat. Prod. Rep. 2019, 36, 476–489. 10.1039/C8NP00053K. 30246844

[ref5] Holton J. M.; Frankel K. A. The Minimum Crystal Size Needed for a Complete Diffraction Data Set. Acta Crystallogr., Sect. D: Struct. Biol. 2010, 66, 393–408. 10.1107/S0907444910007262. PMC2852304 20382993

[ref6] Inokuma Y.; Yoshioka S.; Ariyoshi J.; Arai T.; Hitora Y.; Takada K.; Matsunaga S.; Rissanen K.; Fujita M. X-ray Analysis on the Nanogram to Microgram Scale Using Porous Complexes. Nature 2013, 495, 461–466. 10.1038/nature11990. 23538828

[ref7] Zigon N.; Duplan V.; Wada N.; Fujita M. Crystalline Sponge Method: X-ray Structure Analysis of Small Molecules by Post-Orientation within Porous Crystals–Principle and Proof-of-Concept Studies. Angew. Chem., Int. Ed. 2021, 60, 25204–25222. 10.1002/anie.202106265. 34109717

[ref8] Cheng Y. Single-Particle Cryo-EM—How Did It Get Here and Where Will It Go. Science 2018, 361, 876–880. 10.1126/science.aat4346. 30166484 PMC6460916

[ref9] Gross L. Recent Advances in Submolecular Resolution with Scanning Probe Microscopy. Nat. Chem. 2011, 3, 273–278. 10.1038/nchem.1008. 21430684

[ref10] Jelínek P. High Resolution SPM Imaging of Organic Molecules with Functionalized Tips. J. Phys.: Condens. Matter 2017, 29, 343002 10.1088/1361-648X/aa76c7. 28749786

[ref11] Bian K.; Gerber C.; Heinrich A. J.; Müller D. J.; Scheuring S.; Jiang Y. Scanning Probe Microscopy. Nat. Rev. Methods Primers 2021, 1, 36 10.1038/s43586-021-00033-2.

[ref12] Zhang X.; Zeng Q.; Wang C. On-Surface Single Molecule Synthesis Chemistry: A Promising Bottom-Up Approach towards Functional Surfaces. Nanoscale 2013, 5, 8269–8287. 10.1039/c3nr01611k. 23748971

[ref13] Wang C.; Chi L.; Ciesielski A.; Samorì P. Chemical Synthesis at Surfaces with Atomic Precision: Taming Complexity and Perfection. Angew. Chem., Int. Ed. 2019, 58, 18758–18775. 10.1002/anie.201906645. 31407848

[ref14] Hla S.-W.; Rieder K.-H. STM Control of Chemical Reactions: Single-Molecule Synthesis. Annu. Rev. Phys. Chem. 2003, 54, 307–330. 10.1146/annurev.physchem.54.011002.103852. 12626735

[ref15] Spong J.; Mizes H.; LaComb L. Jr; Dovek M. M.; Frommer J. E.; Foster J. S. Contrast Mechanism for Resolving Organic Molecules with Tunnelling Microscopy. Nature 1989, 338, 137–139. 10.1038/338137a0.

[ref16] Foster J.; Frommer J. Imaging of Liquid Crystals using a Tunnelling Microscope. Nature 1988, 333, 542–545. 10.1038/333542a0.

[ref17] Hansma P. K.; Elings V. B.; Marti O.; Bracker C. E. Scanning Tunneling Microscopy and Atomic Force Microscopy: Application to Biology and Technology. Science 1988, 242, 209–216. 10.1126/science.3051380. 3051380

[ref18] Hamann C.; Woltmann R.; Hong I.-P.; Hauptmann N.; Karan S.; Berndt R. Ultrahigh Vacuum Deposition of Organic Molecules by Electrospray Ionization. Rev. Sci. Instrum. 2011, 82, 033903 10.1063/1.3553010. 21456759

[ref19] Rauschenbach S.; Ternes M.; Harnau L.; Kern K. Mass Spectrometry as a Preparative Tool for the Surface Science of Large Molecules. Annu. Rev. Anal. Chem. 2016, 9, 473–498. 10.1146/annurev-anchem-071015-041633. 27089378

[ref20] Kley C. S.; Dette C.; Rinke G.; Patrick C. E.; Čechal J.; Jung S. J.; Baur M.; Dürr M.; Rauschenbach S.; Giustino F.; Stepanow S.; Kern K. Atomic-Scale Observation of Multiconformational Binding and Energy Level Alignment of Ruthenium-Based Photosensitizers on TiO_2_ Anatase. Nano Lett. 2014, 14, 563–569. 10.1021/nl403717d. 24471471

[ref21] Deng Z.; Thontasen N.; Malinowski N.; Rinke G.; Harnau L.; Rauschenbach S.; Kern K. A Close Look at Proteins: Submolecular Resolution of Two- and Three-Dimensionally Folded Cytochrome C at Surfaces. Nano Lett. 2012, 12, 2452–2458. 10.1021/nl3005385. 22530980

[ref22] Warr D. A.; Perdigão L. M. A.; Pinfold H.; Blohm J.; Stringer D.; Leventis A.; Bronstein H.; Troisi A.; Costantini G. Sequencing Conjugated Polymers by Eye. Sci. Adv. 2018, 4, eaas9543 10.1126/sciadv.aas9543. 29922716 PMC6003723

[ref23] Abb S.; Harnau L.; Gutzler R.; Rauschenbach S.; Kern K. Two-Dimensional Honeycomb Network Through Sequence-Controlled Self-Assembly of Oligopeptides. Nat. Commun. 2016, 7, 10335 10.1038/ncomms10335. 26755352 PMC4729956

[ref24] Rauschenbach S.; Rinke G.; Gutzler R.; Abb S.; Albarghash A.; Le D.; Rahman T. S.; Dürr M.; Harnau L.; Kern K. Two-Dimensional Folding of Polypeptides into Molecular Nanostructures at Surfaces. ACS Nano 2017, 11, 2420–2427. 10.1021/acsnano.6b06145. 28122181

[ref25] Abb S.; Tarrat N.; Cortés J.; Andriyevsky B.; Harnau L.; Schön J. C.; Rauschenbach S.; Kern K. Carbohydrate Self-Assembly at Surfaces: STM Imaging of Sucrose Conformation and Ordering on Cu(100). Angew. Chem., Int. Ed. 2019, 58, 8336–8340. 10.1002/anie.201901340. PMC6771801 31018027

[ref26] Abb S.; Tarrat N.; Cortés J.; Andriyevsky B.; Harnau L.; Schön J. C.; Rauschenbach S.; Kern K. Polymorphism in Carbohydrate Self-Assembly at Surfaces: STM Imaging and Theoretical Modelling of Trehalose on Cu(100). RSC Adv. 2019, 9, 35813–35819. 10.1039/C9RA06764G. 35528101 PMC9074738

[ref27] Wu X.; Delbianco M.; Anggara K.; Michnowicz T.; Pardo-Vargas A.; Bharate P.; Sen S.; Pristl M.; Rauschenbach S.; Schlickum U.; Abb S.; Seeberger P. H.; Kern K. Imaging Single Glycans. Nature 2020, 582, 375–378. 10.1038/s41586-020-2362-1. 32555487

[ref28] Seibel J.; Fittolani G.; Mirhosseini H.; Wu X.; Rauschenbach S.; Anggara K.; Seeberger P. H.; Delbianco M.; Kühne T. D.; Schlickum U.; Kern K. Visualizing Chiral Interactions in Carbohydrates Adsorbed on Au(111) by High-Resolution STM Imaging. Angew. Chem., Int. Ed. 2023, 62, e202305733 10.1002/anie.202305733. 37522820

[ref29] Anggara K.; Sršan L.; Jaroentomeechai T.; Wu X.; Rauschenbach S.; Narimatsu Y.; Clausen H.; Ziegler T.; Miller R. L.; Kern K. Direct Observation of Glycans Bonded to Proteins and Lipids at the Single-Molecule Level. Science 2023, 382, 219–223. 10.1126/science.adh3856. 37824645 PMC7615228

[ref30] Moro S.; Siemons N.; Drury O.; Warr D. A.; Moriarty T. A.; Perdigão L. M. A.; Pearce D.; Moser M.; Hallani R. K.; Parker J.; McCulloch I.; Frost J. M.; Nelson J.; Costantini G. The Effect of Glycol Side Chains on the Assembly and Microstructure of Conjugated Polymers. ACS Nano 2022, 16, 21303–21314. 10.1021/acsnano.2c09464. 36516000 PMC9798861

[ref31] Esser T. K.; Böhning J.; Önür A.; Chinthapalli D. K.; Eriksson L.; Grabarics M.; Fremdling P.; Konijnenberg A.; Makarov A.; Botman A.; Peter C.; Benesch J. L. P.; Robinson C. V.; Gault J.; Baker L.; Bharat T. A. M.; Rauschenbach S. Cryo-EM of Soft-Landed β-Galactosidase: Gas-Phase and Native Structures are Remarkably Similar. Sci. Adv. 2024, 10, eadl4628 10.1126/sciadv.adl4628. 38354247 PMC10866560

[ref32] Rodríguez-Galván A.; Contreras-Torres F. F. Scanning Tunneling Microscopy of Biological Structures: An Elusive Goal for Many Years. Nanomaterials 2022, 12, 3013 10.3390/nano12173013. 36080050 PMC9457988

[ref33] Anggara K.; Zhu Y.; Delbianco M.; Rauschenbach S.; Abb S.; Seeberger P. H.; Kern K. Exploring the Molecular Conformation Space by Soft Molecule–Surface Collision. J. Am. Chem. Soc. 2020, 142, 21420–21427. 10.1021/jacs.0c09933. 33167615 PMC7760097

[ref34] Rosa-Fraile M.; Rodríguez-Granger J.; Haidour-Benamin A.; Cuerva J. M.; Sampedro A. Granadaene: Proposed Structure of the Group B Streptococcus Polyenic Pigment. Appl. Environ. Microbiol. 2006, 72, 6367–6370. 10.1128/AEM.00756-06. 16957264 PMC1563658

[ref35] Paradas M.; Jurado R.; Haidour A.; Rodríguez Granger J.; Sampedro Martínez A.; de la Rosa Fraile M.; Robles R.; Justicia J.; Cuerva J. M. Clarifying the Structure of Granadaene: Total Synthesis of Related Analogue [2]-Granadaene and Confirmation of its Absolute Stereochemistry. Bioorg. Med. Chem. 2012, 20, 6655–6661. 10.1016/j.bmc.2012.09.017. 23043725

[ref36] Whidbey C.; Harrell M. I.; Burnside K.; Ngo L.; Becraft A. K.; Iyer L. M.; Aravind L.; Hitti J.; Adams Waldorf K. M.; Rajagopal L. A Hemolytic Pigment of Group B Streptococcus Allows Bacterial Penetration of Human Placenta. J. Exp. Med. 2013, 210, 1265–1281. 10.1084/jem.20122753. 23712433 PMC3674703

[ref37] Boldenow E.; Gendrin C.; Ngo L.; Bierle C.; Vornhagen J.; Coleman M.; Merillat S.; Armistead B.; Whidbey C.; Alishetti V.; Santana-Ufret V.; Ogle J.; Gough M.; Srinouanprachanh S.; MacDonald J. W.; Bammler T. K.; Bansal A.; Liggitt H. D.; Rajagopal L.; Adams Waldorf K. M. Group B *Streptococcus* Circumvents Neutrophils and Neutrophil Extracellular Traps during Amniotic Cavity Invasion and Preterm Labor. Sci. Immunol. 2016, 1, eaah4576 10.1126/sciimmunol.aah4576. 27819066 PMC5089172

[ref38] Siemens N.; Oehmcke-Hecht S.; Hoßmann J.; Skorka S. B.; Nijhuis R. H. T.; Ruppen C.; Skrede S.; Rohde M.; Schultz D.; Lalk M.; Itzek A.; Pieper D. H.; van den Bout C. J.; Claas E. C. J.; Kuijper E. J.; Mauritz R.; Sendi P.; Wunderink H. F.; Norrby-Teglund A. Prothrombotic and Proinflammatory Activities of the β-Hemolytic Group B Streptococcal Pigment. J. Innate Immun. 2020, 12, 291–303. 10.1159/000504002. 31743913 PMC7383282

[ref39] Armistead B.; Quach P.; Snyder J. M.; Santana-Ufret V.; Furuta A.; Brokaw A.; Rajagopal L. Hemolytic Membrane Vesicles of Group B Streptococcus Promote Infection. J. Infect. Dis. 2021, 223, 1488–1496. 10.1093/infdis/jiaa548. 32861213 PMC8064051

[ref40] Zangwill K. M.; Schuchat A.; Wenger J. D. Group B Streptococcal Disease in the United States, 1990: Report from a Multistate Active Surveillance System. MMWR CDC Surveill Summ. 1992, 41, 25–32. 1470102

[ref41] Francois Watkins L. K.; McGee L.; Schrag S. J.; Beall B.; Jain J. H.; Pondo T.; Farley M. M.; Harrison L. H.; Zansky S. M.; Baumbach J.; Lynfield R.; Snippes Vagnone P.; Miller L. A.; Schaffner W.; Thomas A. R.; Watt J. P.; Petit S.; Langley G. E. Epidemiology of Invasive Group B Streptococcal Infections Among Nonpregnant Adults in the United States, 2008–2016. JAMA Int. Med. 2019, 179, 479–488. 10.1001/jamainternmed.2018.7269. PMC6450309 30776079

[ref42] Sendi P.; Johansson L.; Norrby-Teglund A. Invasive Group B Streptococcal Disease in Non-Pregnant Adults: A Review with Emphasis on Skin and Soft-Tissue Infections. Infection 2008, 36, 100–111. 10.1007/s15010-007-7251-0. 18193384

[ref43] Lawn J. E.; Bianchi-Jassir F.; Russell N. J.; Kohli-Lynch M.; Tann C. J.; Hall J.; Madrid L.; Baker C. J.; Bartlett L.; Cutland C.; Gravett M. G.; Heath P. T.; Ip M.; Le Doare K.; Madhi S. A.; Rubens C. E.; Saha S. K.; Schrag S.; Sobanjo-ter Meulen A.; Vekemans J.; Seale A. C. Group B Streptococcal Disease Worldwide for Pregnant Women, Stillbirths, and Children: Why, What, and How to Undertake Estimates?. Clin. Infect. Dis. 2017, 65, S89–S99. 10.1093/cid/cix653. 29117323 PMC5850012

[ref44] Seale A. C.; Bianchi-Jassir F.; Russell N. J.; Kohli-Lynch M.; Tann C. J.; Hall J.; Madrid L.; Blencowe H.; Cousens S.; Baker C. J.; Bartlett L.; Cutland C.; Gravett M. G.; Heath P. T.; Ip M.; Le Doare K.; Madhi S. A.; Rubens C. E.; Saha S. K.; Schrag S. J.; Sobanjo-ter Meulen A.; Vekemans J.; Lawn J. E. Estimates of the Burden of Group B Streptococcal Disease Worldwide for Pregnant Women, Stillbirths, and Children. Clin. Infect. Dis. 2017, 65, S200–S219. 10.1093/cid/cix664. 29117332 PMC5849940

[ref45] Gonçalves B. P; Procter S. R; Paul P.; Chandna J.; Lewin A.; Seedat F.; Koukounari A.; Dangor Z.; Leahy S.; Santhanam S.; John H. B; Bramugy J.; Bardají A.; Abubakar A.; Nasambu C.; Libster R.; Sánchez Yanotti C.; Horváth-Puhó E.; Sørensen H. T; van de Beek D.; Bijlsma M. W; Gardner W. M; Kassebaum N.; Trotter C.; Bassat Q.; Madhi S. A; Lambach P.; Jit M.; Lawn J. E; Søgaard K. K.; van Kassel M. N.; Snoek L.; de Gier B.; van der Ende A.; Hahne S. J M; Harden L. M.; Ghoor A.; Mbatha S.; Lowick S.; Laughton B.; Jaye T.; Lala S. G; Sithole P.; Msayi J.; Kumalo N.; Msibi T. N.; Arumugam A.; Murugesan N.; Rajendraprasad N.; Priya M.; Mabrouk A.; Katana P. V.; Mwangome E.; Newton C. R.; Mucasse H.; Aerts C.; Massora S.; Medina V.; Rojas A.; Amado D.; Llapur C. J.; Hossain A. K. M. T.; Rahman Q. S.-u.; Ip M.; Seale A.; Heath P. T.; Le Doare K.; Khalil A.; Schrag S. J.; Sobanjo-ter Meulen A.; Mason E.; Blau D. M; El Arifeen S.; Assefa N.; Onyango D.; Sow S. O.; Mandomando I.; Ogbuanu I.; Kotloff K. L.; Scott J. A. G.; Gurley E. S.; Barr B. A. T.; Mahtab S. Group B Streptococcus Infection During Pregnancy and Infancy: Estimates of Regional and Global Burden. Lancet Glob. Health 2022, 10, e807–e819. 10.1016/S2214-109X(22)00093-6. 35490693 PMC9090904

[ref46] Armistead B.; Herrero-Foncubierta P.; Coleman M.; Quach P.; Whidbey C.; Justicia J.; Tapia R.; Casares R.; Millán A.; Haidour A.; Granger J. R.; Vornhagen J.; Santana-Ufret V.; Merillat S.; Adams Waldorf K.; Cuerva J. M.; Rajagopal L. Lipid Analogs Reveal Features Critical for Hemolysis and Diminish Granadaene Mediated Group B Streptococcus Infection. Nat. Commun. 2020, 11, 1502 10.1038/s41467-020-15282-0. 32198389 PMC7083881

[ref47] Furuta A.; Coleman M.; Casares R.; Seepersaud R.; Orvis A.; Brokaw A.; Quach P.; Nguyen S.; Sweeney E.; Sharma K.; Wallen G.; Sanghavi R.; Mateos-Gil J.; Cuerva J. M.; Millán A.; Rajagopal L. CD1 and iNKT Cells Mediate Immune Responses against the GBS Hemolytic Lipid Toxin Induced by a Non-Toxic Analog. PLoS Pathog. 2023, 19, e1011490 10.1371/journal.ppat.1011490. 37384812 PMC10337943

[ref48] Cao N.; Yang B.; Riss A.; Rosen J.; Björk J.; Barth J. V. On-Surface Synthesis of Enetriynes. Nat. Commun. 2023, 14, 1255 10.1038/s41467-023-36828-y. 36878914 PMC9988975

[ref49] Pracht P.; Bohle F.; Grimme S. Automated Exploration of the Low-Energy Chemical Space with Fast Quantum Chemical Methods. Phys. Chem. Chem. Phys. 2020, 22, 7169–7192. 10.1039/C9CP06869D. 32073075

[ref50] Mendieta-Moreno J. I.; Walker R. C.; Lewis J. P.; Gómez-Puertas P.; Mendieta J.; Ortega J. An Efficient Local-Orbital DFT QM/MM Method for Biomolecular Systems. J. Chem. Theory Comput. 2014, 10, 2185–2193. 10.1021/ct500033w. 26580543

[ref51] Horcas I.; Fernández R.; Gómez-Rodríguez J. M.; Colchero J.; Gómez-Herrero J.; Baro A. M. WSXM: A Software for Scanning Probe Microscopy and a Tool for Nanotechnology. Rev. Sci. Instrum. 2007, 78, 013705 10.1063/1.2432410. 17503926

[ref52] Frisch M. J.; Trucks G. W.; Schlegel H. B.; Scuseria G. E.; Robb M. A.; Cheeseman J. R.; Scalmani G.; Barone V.; Petersson G. A.; Nakatsuji H.; Li X.; Caricato M.; Marenich A. V.; Bloino J.; Janesko B. G.; Gomperts R.; Mennucci B.; Hratchian H. P.; Ortiz J. V.; Izmaylov A. F.; Sonnenberg J. L.; Williams-Young D.; Ding F.; Lipparini F.; Egidi F.; Goings J.; Peng B.; Petrone A.; Henderson T.; Ranasinghe D.; Zakrzewski V. G.; Gao J.; Rega N.; Zheng G.; Liang W.; Hada M.; Ehara M.; Toyota K.; Fukuda R.; Hasegawa J.; Ishida M.; Nakajima T.; Honda Y.; Kitao O.; Nakai H.; Vreven T.; Throssell K.; Montgomery J. A. Jr.; Peralta J. E.; Ogliaro F.; Bearpark M. J.; Heyd J. J.; Brothers E. N.; Kudin K. N.; Staroverov V. N.; Keith T. A.; Kobayashi R.; Normand J.; Raghavachari K.; Rendell A. P.; Burant J. C.; Iyengar S. S.; Tomasi J.; Cossi M.; Millam J. M.; Klene M.; Adamo C.; Cammi R.; Ochterski J. W.; Martin R. L.; Morokuma K.; Farkas O.; Foresman J. B.; Fox D. J. Gaussian 16, Revision C.01; Gaussian, Inc.: Wallingford, CT, 2016.

[ref53] Lewis J. P.; Jelinek P.; Ortega J.; Demkov A. A.; Trabada D. G.; Haycock B.; Wang H.; Adams G.; Tomfohr J. K.; Abad E.; Wang H.; Drabold D. A. Advances and Applications in the FIREBALL Ab Initio Tight-Binding Molecular-Dynamics Formalism. Phys. Status Solidi B 2011, 248, 1989–2007. 10.1002/pssb.201147259.

[ref54] Heinz H.; Lin T.-J.; Kishore Mishra R.; Emami F. S. Thermodynamically Consistent Force Fields for the Assembly of Inorganic, Organic, and Biological Nanostructures: The INTERFACE Force Field. Langmuir 2013, 29, 1754–1765. 10.1021/la3038846. 23276161

