# Supplementary material for: Single-Molecule Identification of the Isomers of a Lipidic Antibody Activator
Source: J Phys Chem Lett. 2024 Jun 27;15(27):6935–42. doi: 10.1021/acs.jpclett.4c00164 (PMC11247479; doi:10.1021/acs.jpclett.4c00164)
Supplement: Supplementary file 1 — jz4c00164_si_001.pdf [file jz4c00164_si_001.pdf]

Single-Molecule Identification of the Isomers of a Lipidic Antibody Activator

Benjamin Mallada[1,2‡], Federico Villalobos[3‡], Beatriz Donoso[3], Raquel Casares[3], Giovanna Longhi[4], Jesús I. Mendieta-Moreno[5], Alejandro Jiménez-Martín[1,2,6], Ali Haïdour[7], Ravin Seepersaud[8], Lakshmi Rajagopal[8,9,10], Bruno de la Torre [2], Alba Millán[3]*, Juan M. Cuerva[3]**

[1] Institute of Physics, Czech Academy of Sciences, 16200 Prague, Czech Republic.

[2] Regional Centre of Advanced Technologies and Materials, Czech Advanced Technology and Research Institute (CATRIN), Palacký University Olomouc, 78371 Olomouc, Czech Republic.

[3] Departamento de Química Orgánica, Unidad de Excelencia de Química Aplicada a la Biomedicina y Medioambiente, C. U. Fuentenueva, Universidad de Granada, 18071, Spain.

[4] Dipartimento di Medicina Molecolare e Traslazionale, Universitá di Brescia, Viale Europa 11, 25121 Brescia, Italy

[5] Instituto de Ciencia de Materiales de Madrid (ICMM), CSIC, 28049, Madrid, España

[6] Faculty of Nuclear Sciences and Physical Engineering, Czech Technical University, 11519 Prague, Czech Republic

[7] Unidad de Resonancia Magnética Nuclear, Centro de Instrumentación Científica, Universidad de Granada, Paseo Juan Osorio S/N, 18071 Granada, Spain

[8] Center for Global Infectious Disease Research, Seattle Children's Research Institute, Seattle, Washington, USA

[9] Department of Global Health, University of Washington, Seattle, Washington, USA

[10] Department of Pediatrics, University of Washington, Seattle, Washington, USA

E-mails: bruno.de@upol.cz, amillan@ugr.es; jmcuerva@ugr.es

Table of contents

1. *General Details* S3
2. *Synthetic procedures* S5
3. *^{1}H-NMR and HRMS spectra of RP4* S6
4. *On-Surface characterization* S8
5. *Computational details* S10
6. *References* S16

1. General Details

In solution synthesis

Unless otherwise stated, all reagents and solvents were purchased from commercial sources and used without further purification. Anhydrous THF was freshly distilled over Na/benzophenone. Flash column chromatography was carried out using silica gel 60 (40-63 µm) as the stationary phase. Analytical TLC was performed on aluminium sheets coated with silica gel with fluorescent indicator UV254 and observed under UV light (254 nm) and/or stained with phosphomolybdic acid (5% methanol solution). All ^{1}H and ^{13}C NMR spectra were recorded on Bruker Avance Neo (400 MHz or 500 MHz) spectrometers at a constant temperature of 298 K. Chemical shifts are reported in ppm and referenced to residual solvent: $CHCl_3$ (7.27 and 77.0 ppm for ^{1}H and ^{13}C respectively), CH_3OH (3.31 and 49.0 ppm for ^{1}H and ^{13}C respectively). Coupling constants (J) are reported in Hertz (Hz). Multiplicities are abbreviated as follow: s = singlet, br s = broad singlet, d = doublet, t =triplet m = multiplet, dd = doublet of doublets, td = triplet of doublets, ddd = doublet of doublet of doublets, dt = doublet of triplets. Proton assignment was carried out by 2D NMR experiments: COSY, HSQC and HMBC where possible. Assignment of the ^{13}C NMR multiplicities was accomplished by DEPT techniques. ESI-TOF mass spectra were recorded in a Waters Xevo G2-XS QTof. **RP4** was prepared according to the literature[S1] with some modifications detailed in the synthetic section.

SPM experiments

The experiments were conducted within an ultra-high vacuum (UHV) environment, where the base pressure was maintained below 5 x 10^{-10} mbar. The setup included a low-temperature scanning tunneling microscope (Createc GmbH) operating at 4.2 K. Imaging was performed using a Pt/Ir tip with the bias voltage applied to the sample. Metallic tips with atomically sharp apexes were achieved through controlled indentations on the exposed surface. The Au(111) was prepared via standard cycles of Ar+ sputtering and annealing. The electrospray depositions were carried out using a commercial system (MolecularSpray Ltd) outfitted with several pumping stages. Our ESD setup run in positive mode, that is, a positive bias is applied to the emitter. The setup was linked to the UHV preparation chamber. **RP4** was dissolved in methanol to form a solution with concentration of 2 mg/mL. The deposition was performed onto the sample at ambient temperature. During the spray deposition, the pressure in the chamber was less than 1x10^{-7} mbar. Typically, the voltages applied to the capillary ranged between 2-2.3 kV, with necessary adjustments to maintain spray stability while keeping an approximate pumping rate of 60 µL/h for 60 min. After deposition, the sample was immediately transferred to the analysis chamber in UHV and cooled down to 4.2K.

All data were subject to standard processes using the WSxM software[S2] without any filtering or smoothing. Approximately, more than 20 **RP4** molecules were considered in dozens of STM overviews of 50 x 50 nm^2.

The molecular manipulation to separate **RP4** from the conglomerated islands was performed by scanning in constant current mode a conglomerate with a bias voltage of 1 mV and a tunneling current of 10 pA. After every manipulation event, the frame was rescanned in constant current mode with a bias voltage of 500 mV and a tunneling current of 10 pA.

2. Synthetic procedures

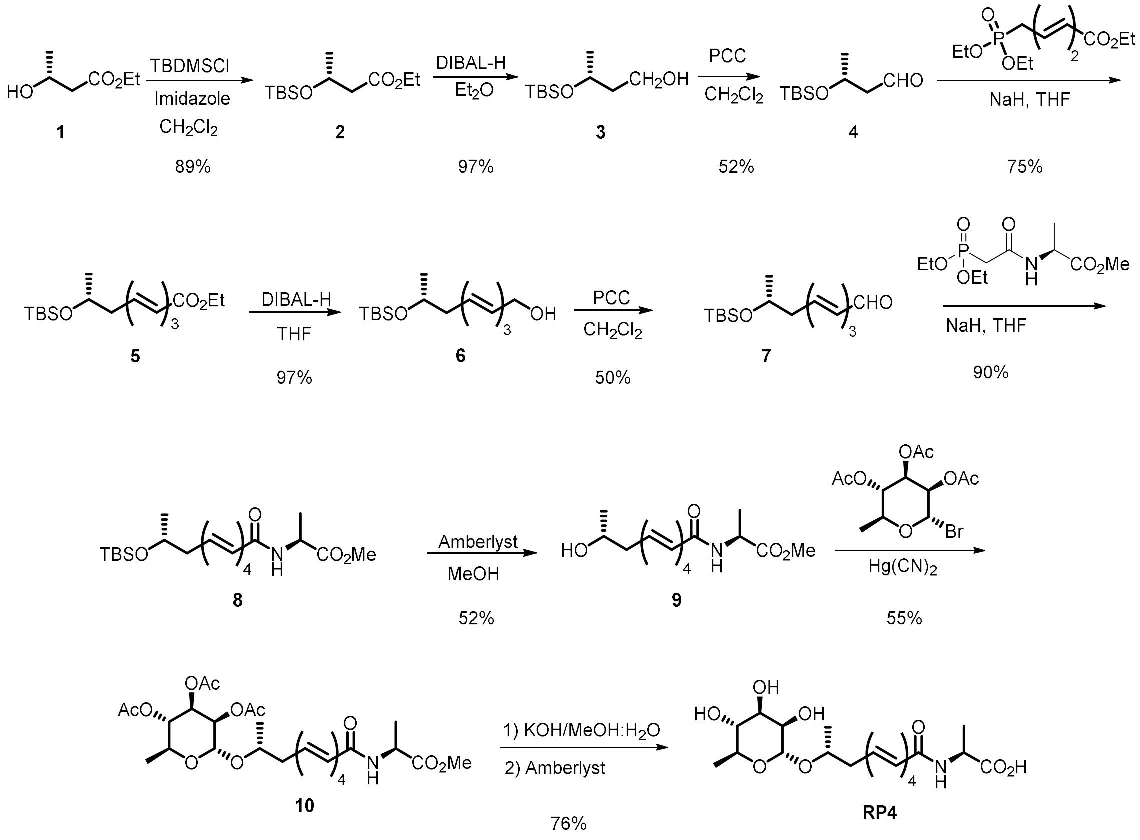

Scheme S1. Synthesis of **RP4**.

The synthesis and characterization of **RP4** and all compounds has been described previously,[S1] but aldehydes **4** and **7** have been prepared using another procedure.

General procedure for the synthesis of aldehydes[S3]

To a solution of the corresponding alcohol (1 equiv.) in CH_2Cl_2 (0.2M), pyridinium chlorochromate (PCC, 2 equiv.) and celite (same quantity as PCC) were added, and the mixture was stirred at room temperature for 2 h. Afterwards the residue was filtered through a Celite®/silica gel plug, and the corresponding aldehyde was obtained without further purification.

Synthesis of **4.** Aldehyde **4** was prepared from alcohol **3** (2g, 9.8mmol) in 52% yield. Its ^1H and ^{13}C NMR spectra matched with those previously described.[S1]

Synthesis of **7.** From **6** (2g, 7.07mmol), aldehyde **7** was synthesized in 50% yield. Its ^1H and ^{13}C NMR spectra matched with those previously described.[S1]

3. ^{1}H-NMR and high-resolution mass spectra of RP4

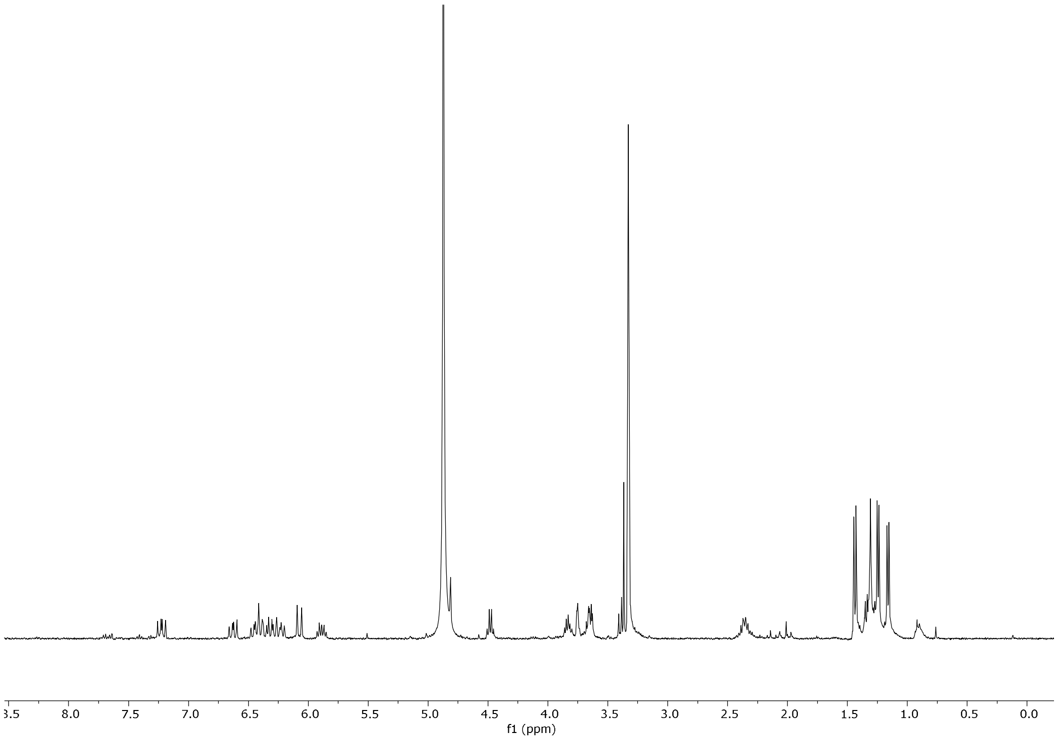

Figure S1. ^{1}H-NMR (500 MHz, MeOH-d_4) spectrum of **RP4**. Data of the major isomer: δ (ppm) 7.18 (dd, *J* = 15.1, 11.0 Hz, 1H), 6.59 (dd, *J* = 14.8, 10.9 Hz, 1H), 6.44–6.32 (m, 2H), 6.27 (dd, *J* = 14.9, 10.9 Hz, 1H), 6.21 (dd, *J* = 15.2, 10.6 Hz, 1H), 6.07 (d, *J* = 14.8 Hz, 1H), 5.86 (dt, *J* = 14.8, 7.2 Hz, 1H), 4.48 (q, *J* = 7.6 Hz, 1H), 3.83 (m, 1H), 3.74 (bs, 1H), 3.68–3.61 (m, 2H), 3.37 (t, *J* = 9.5 Hz, 2H), 2.45–2.28 (m, 2H), 1.38 (d, *J* = 7.1 Hz, 3H), 1.23 (d, *J* = 6.4 Hz, 3H), 1.15 (d, *J* = 6.1 Hz, 3H)

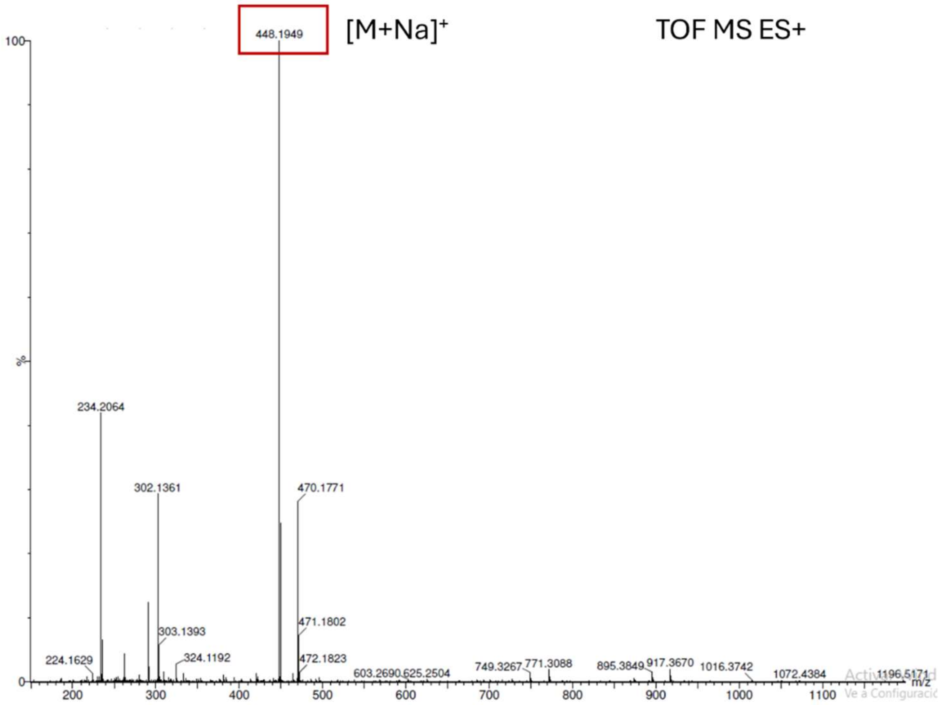

Figure S2. High-resolution mass spectra (electrospray ionization, positive mode) of a sample of **RP4**. The red label corresponds to the $[M+Na]^+$ ion.

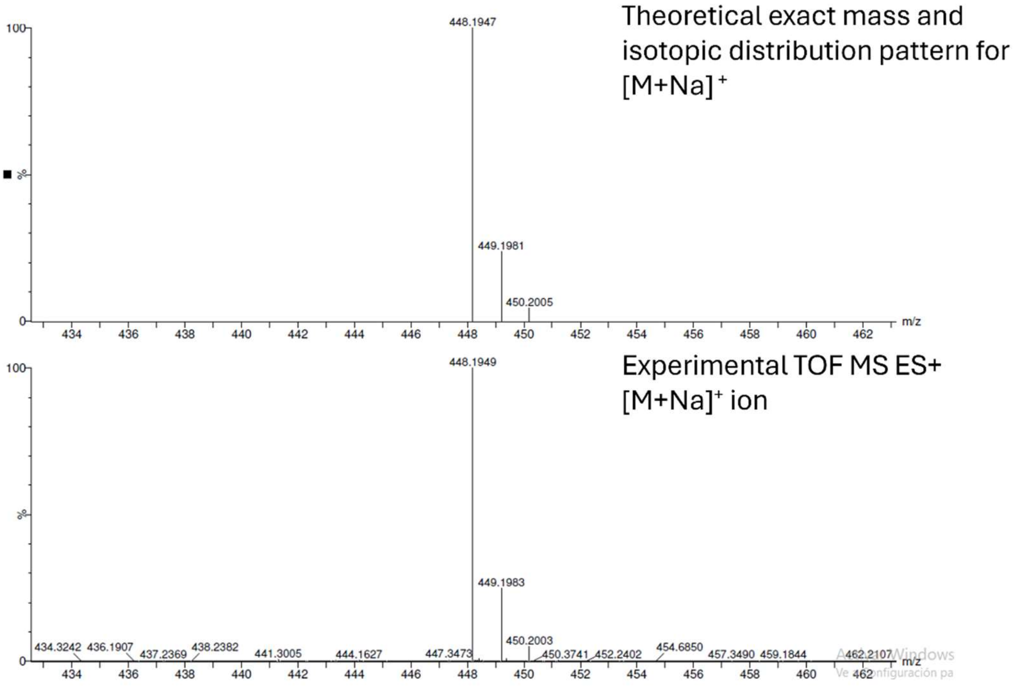

Figure S3. Theoretical (top) and experimental (bottom) mass and isotopic distribution pattern of $[M+Na]^+$ ion of **RP4**. ($C_{21}H_{31}NO_8Na$ calcd: 448.1947; found: 448.1949).

4. On-Surface characterization

Manipulation experiments

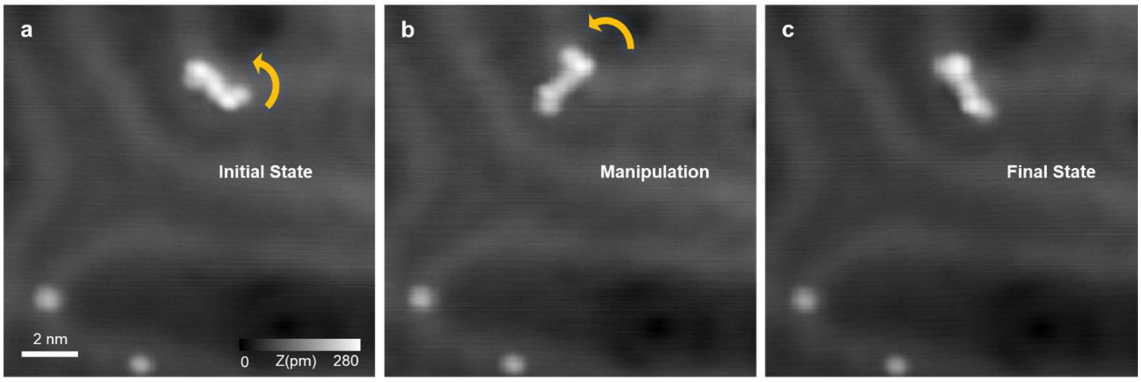

Figure S4. Sequence of STM images displaying a tip-induced manipulation on a single **RP4** molecule from an initial state a) to a final state c) through a sequence of rotations. Scanning parameters: **a)** Vs = 51 mV, I = 10 pA, **b)** Vs = 51 mV, I = 10 pA, **c)** Vs = 51 mV, I = 10 pA.

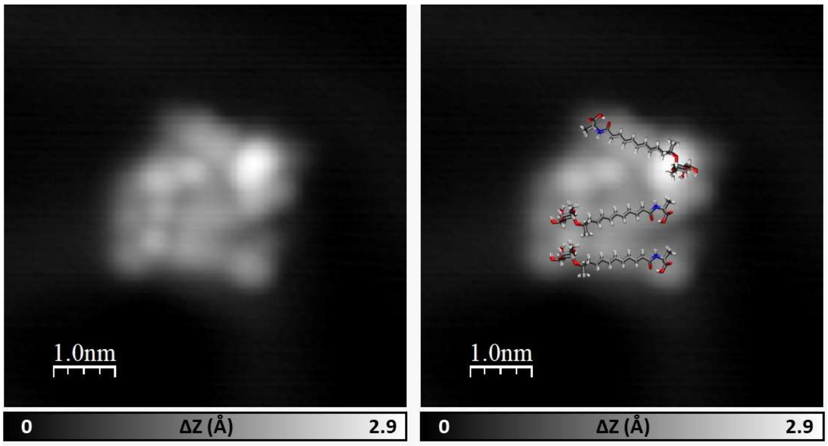

Figure S5: STM topography image of a molecular aggregate with up to 3 intact molecules of **RP4**.

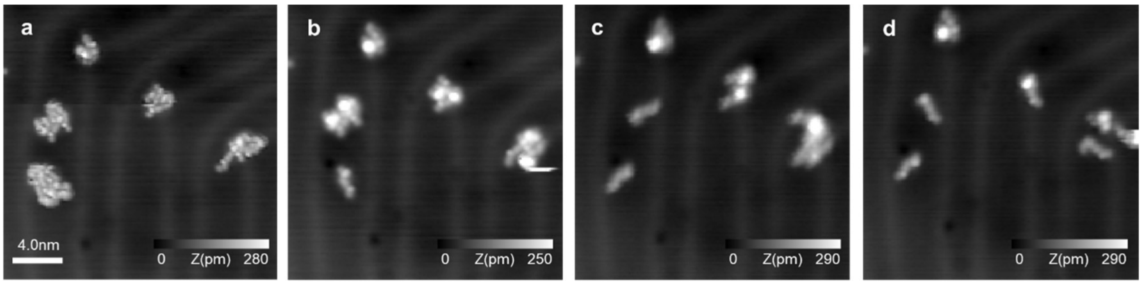

Figure S6. Constant current STM images of the separation of **RP4** clusters by tip manipulation. Scanning parameters: **a)** Vs = 501 mV, I = 10 pA, **b)** Vs = 501 mV, I = 10 pA, **c)** Vs = 501 mV, I = 10 pA, **d)** Vs = 501 mV, I = 10 pA.

Height measurements of isomers.

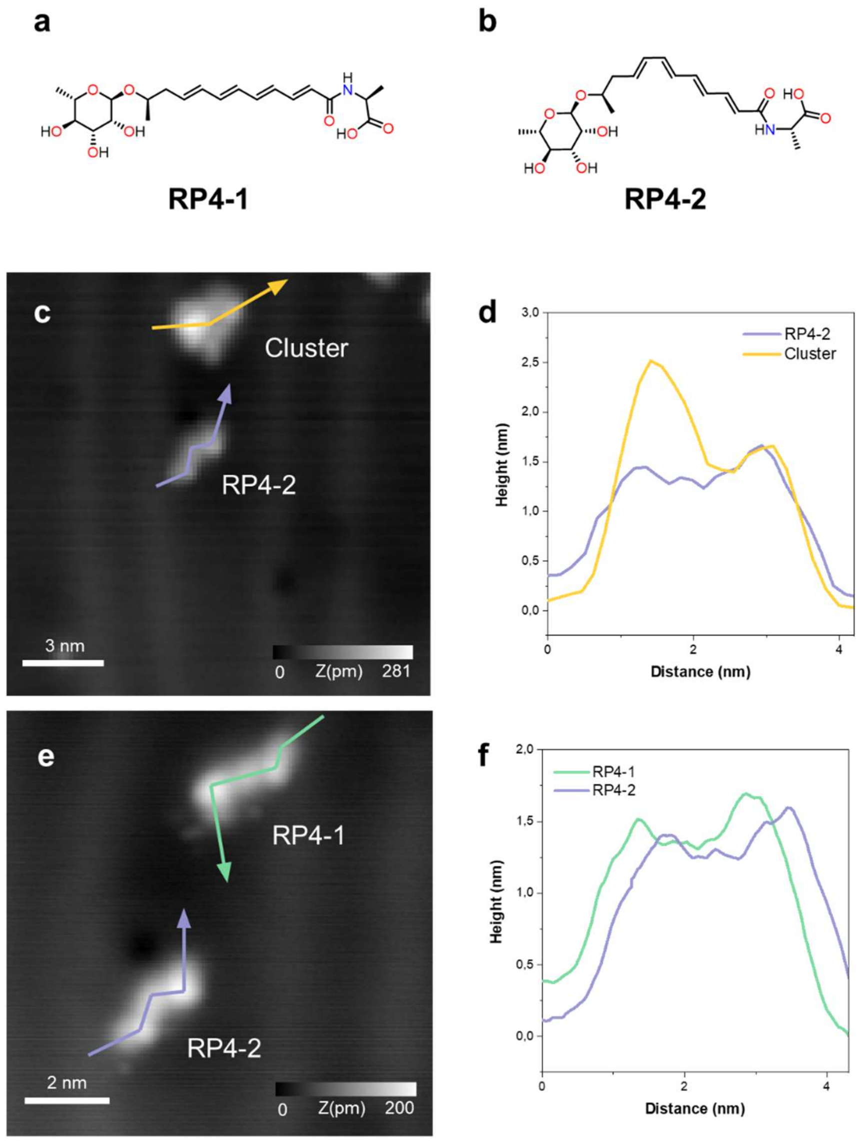

Figure S7. Height measurements of **RP4** isomers. **(a, b)** Chemical models of the visualized RP4 isomers. **(c)** Constant-current STM images of cluster and isomer 2. **(e)** Constant-current STM images of isomer 1 and isomer 2. **(d, f)** Height measurements of **RP4** isomers. Arrow depicts the followed path to measure the height on the STM images. Scanning parameters: **c)** Vs = 501 mV, I = 10 pA, **e)** Vs = 501 mV, I = 10 pA.

5. Computational details

5.1. Conformational analysis

In the following, we aim at obtaining structures compatible with STM images starting from an ensemble of possible conformers. As a first step, we performed a conformational search of the amino-acid end residue as represented in fig. S8A and of the rhamnose group as represented in Figure S8B. The results indicate that, as expected, the preferred amino geometry maintains the OC-NH dihedral angle at about 180° (trans); concerning the sugar ring, the chair conformation with the bulky group O-$CHCH_3$-CH_2-CH_3 in axial orientation is energetically favoured (mantaining the methyl and two out of three hydroxyl groups equatorial).

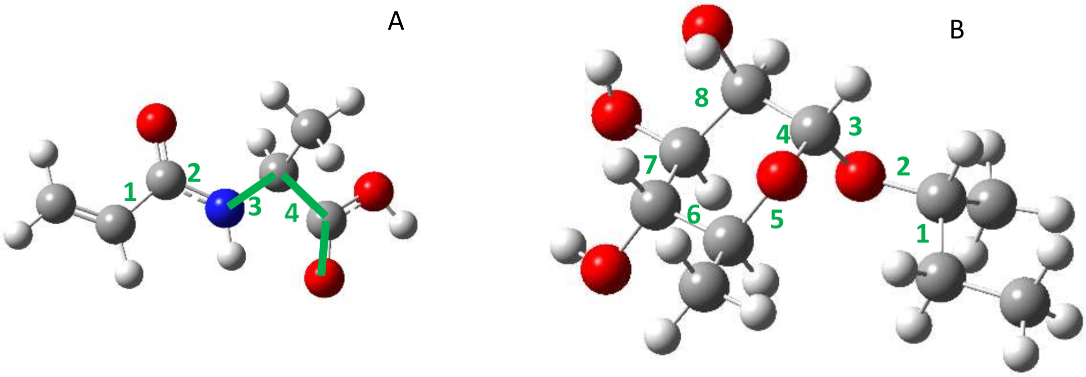

Figure S8. Models for aminoacid (A) and rhamnose groups (B) of **RP4** for first conformational search. Numbers indicate dihedral angles reported in Table S1 and S2

Table S1. Results from conformational search in vacuo of aminoacid group at M06/tzvp level. Angles reported in the table are defined in figure S8 and are in degrees. Labels 'trans' or 'cis' refer to peptide bond (dihedral angle 2).

	Energy Kcal/mol	Stat.weigth	1	2	3	4
trans1	0.00	*61.3%*	-180	177	-159	-10
trans2	0.38	*32.4%*	-179	-173	-79	-117
trans3	1.69	*3.5%*	28	179	-157	-8
trans4	1.85	*2.7%*	-29	176	-160	-12
cis1	4.89	*0.0%*	-175	6	-164	-16
cis2	5.79	*0.0%*	177	-11	-65	-27
cis3	5.83	*0.0%*	176	-10	-62	147

Table S2. Results from conformational search in vacuo of **RP4** at M06/tzvp level. Angles reported in the table are defined in figure S8 and are in degrees. Conformers labelled 'ax'correspond to a chair with O-$CHCH_3$-CH_2-CH_3 group in axial orientation, just two equatorial conformers are reported, other equatorial structures are at higher energy.

	Energy Kcal/mol	Stat.weigth	1	2	3	4	5	6	7	8
ax1	0.00	*59.8%*	-61	167	-62	-59	-61	55	-53	52
ax2	0.98	*11.3%*	-178	155	-66	-58	-61	55	-52	52
ax3	1.00	*11.1%*	61	101	-70	-61	-60	55	-53	53
ax4	1.13	*8.9%*	64	158	-64	-58	-61	55	-52	52
ax5	1.36	*6.0%*	-179	87	-77	-60	-62	55	-53	53
ax6	2.05	*1.9%*	-51	-65	-80	-58	-63	55	-52	52
ax7	2.68	*0.6%*	-88	74	-86	-57	-63	55	-52	52
ax8	3.06	*0.3%*	-168	-72	-85	-58	-62	55	-52	53
eq1	3.91	*0.1%*	-64	143	-81	178	61	-53	49	-50
eq2	4.97	*0.0%*	-179	140	-79	178	61	-53	49	-50

As regards the whole molecule, a completely unbiased conformational search in vacuo with the CREST tool[S4] gives a prevalence of distorted structures in which the sugar and amino-acid terminus are H-bonded, with a curved polyene chain that does not correspond to observation. A quite demanding treatment of the problem should be considered, to properly treat an Au surface at DFT level: the surface should be quite extended to host the elongated molecule, and then one should consider systematically the high number of possible conformers in order the test how the surface stabilizes the various structures. To have an idea of all conformational possibilities we report in table S3 the results of the conformational search in vacuo, keeping the chain trans-planar, while leaving all possibilities discussed above for the head and tail groups. After CREST conformational search, structures have been optimized at M06/tzvp level with Gaussian16 package.[S5]

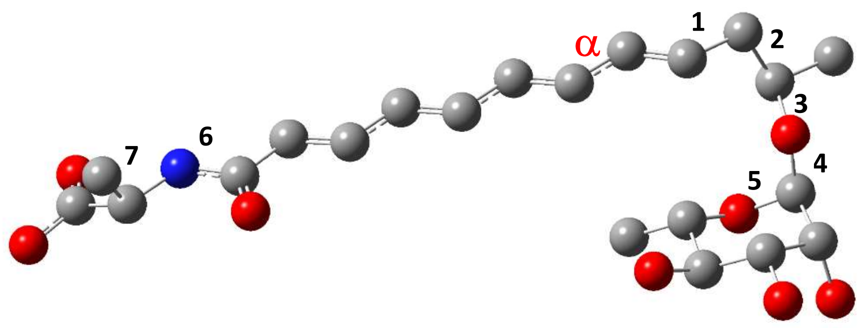

Figure S9. Definition of the principal dihedral angles used in table S3 and S4 to characterize conformers.

Table S3. Results from conformational search in vacuo constraining the polyene chain in all-*trans* configuration. Structures have been optimized with no restrain, except for the polyene chain, at M06/tzvp level. Angles reported in the table are defined in figure S9 and are in degrees.

	Energy Kcal/mol	Stat. Weight	1	2	3	4	5	6	7
1	0.00	*49.0%*	99	-61	161	-58	-59	177	-158
2	0.14	*38.7%*	-98	-56	156	-63	-58	177	-158
3	1.56	*3.5%*	120	63	159	-64	-59	177	-159
4	1.64	*3.1%*	-120	-173	158	-64	-58	177	-158
5	1.84	*2.2%*	-103	56	124	-70	-58	177	-159
6	2.20	*1.2%*	109	-178	156	-65	-58	177	-158
7	2.31	*1.0%*	104	-180	84	-78	-60	177	-158
8	2.57	*0.6%*	118	-47	-64	-86	-58	178	-159
9	3.23	*0.2%*	120	63	160	-63	-59	172	-70
10	3.66	*0.1%*	-119	-53	-69	-82	-58	177	-158
11	3.66	*0.1%*	-120	-176	89	-73	-65	177	-159
12	3.83	*0.1%*	-120	-173	158	-64	-59	-171	-79
13	4.12	*0.0%*	103	-180	90	-73	-65	177	-158
14	4.42	*0.0%*	-119	-59	110	-149	-61	177	-158
15	4.46	*0.0%*	95	-65	107	-149	-60	177	-159
16	5.33	*0.0%*	-122	-53	-66	-80	-58	172	-71
17	5.59	*0.0%*	97	-60	161	-57	-59	167	69
18	5.72	*0.0%*	-99	65	-173	-67	180	177	-158
19	5.81	*0.0%*	126	-90	57	-87	-62	177	-158
20	5.86	*0.0%*	105	-179	85	-77	-60	-175	56
21	5.89	*0.0%*	-100	-57	156	-63	-58	167	69
22	6.09	*0.0%*	-117	-59	110	-150	-61	172	-70
23	7.48	*0.0%*	-121	-174	157	-65	-59	167	69
24	7.65	*0.0%*	102	-180	89	-74	-65	-175	56
25	8.25	*0.0%*	126	-89	57	-88	-62	175	-65
26	9.13	*0.0%*	-121	-173	157	-65	-58	-27	77
27	9.35	*0.0%*	-119	-175	86	-77	-60	-26	77
28	9.65	*0.0%*	123	-87	50	-172	-68	-180	-155
29	9.81	*0.0%*	103	-180	89	-74	-65	167	69
30	11.25	*0.0%*	-118	-163	-70	-83	-58	-26	77
31	13.11	*0.0%*	-119	-176	138	-78	178	-27	77
32	13.18	*0.0%*	-120	-168	-78	-90	179	167	69
33	13.25	*0.0%*	-118	-173	104	-70	178	-26	77
34	13.71	*0.0%*	-119	-173	158	-64	-59	-3	-122
35	14.08	*0.0%*	180	-52	77	173	-65	177	-120
36	14.11	*0.0%*	-119	-178	126	50	174	167	69

Two most populated conformers have been identified which are partially folded, with the rhamnose parallel to the polyene chain, such that they do not really fit the STM images (the first one is reported in Figure S10, top). In general, it is expected that interaction with Au surface[S6] maintains some characteristic of the molecular shape, however, after the manipulations used also to separate molecules, we find that the Au surface stabilizes the structures that are more elongated in comparison to the most populated conformers found in vacuo: it is likely that the molecular manipulation with STM tip unfolds the sugar ring away from the polyene chain. One may notice that the STM images present a bright protrusion possibly in correspondence with the rhamnose ring and its methyl, in fact similar situations may be found in the literature:[S7,S8] protrusions may be associated to sugar rings with "vertical" orientation with respect to the Au surface; on the contrary the polyene chain of **RP4** seems parallel to the surface.

Guided by these considerations suggested by the experimental image, we have adjusted the dihedral angles in correspondence with of the bridge between the sugar ring and the polyene chain, since our aim was just to pinpoint possible structures that may well fit the STM images. The **RP4** structure thus obtained (carbon atoms reported in green) is also compared to one of the most populated conformer obtained in vacuo (carbon atoms reported in light blue) in figure S10: the acid terminus is expected to easily rotate, such to easily get in contact with the Au surface; most importantly, to make the sugar get in contact with the surface instead of being folded towards the polyene chain, three dihedral angles should cooperatively rotate. Without pretending that the one represented in the figure is the unique correct structure, the picture is in any case a plausible one.

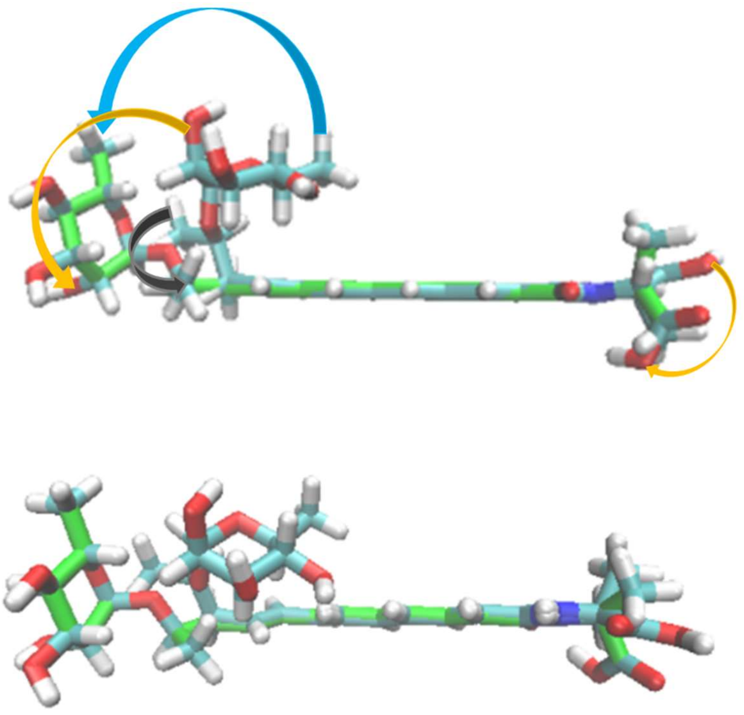

Figure S10. Superposition of the structure obtained in order to be compatible with the STM image (carbon atoms reported in green) with one of the most populated conformers obtained in

vacuo (carbon atoms reported in light blue) Top: **isomer 1** with trans-planar polyene chain. Bottom: **isomer 2** treated with the same procedure.

Once we fixed the dihedral angles necessary to have a good rahmnose-polyene reciprocal orientation, we obtain a good fit with the STM image as shown in figure S11. We report two views of such structure, in order to evidence that it is flat, with good interaction with the metal surface through the polyene backbone and through the hydroxils and acidic terminus pointing towards the metal.

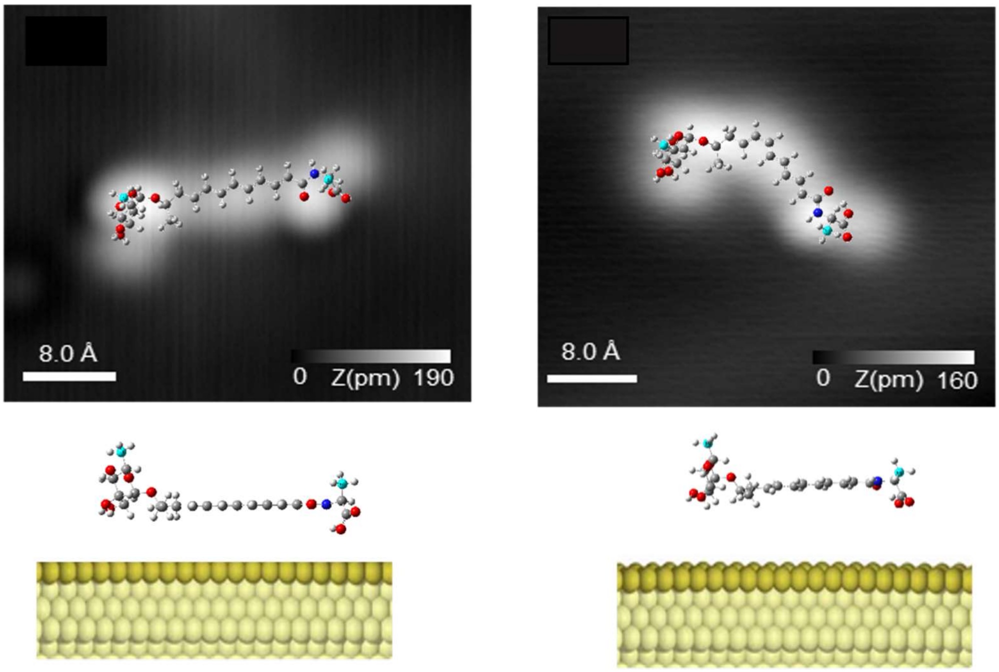

Figure S11. Visualization of **RP4** isomers. Constant-current STM images of the isomers **RP4-1**, on the left and **RP4-2**, on the right. DFT structures compatible with the observed images are superimposed as viewed from top and from the side (parallel to a sketched Au surface). Rhamnose group and amino acid group are clearly distinguishable at the two ends of the polyene chain. Light blue indicates the carbon atoms further away from the surface which are indicative of expected bright protrusions, due to the vertical orientation of the rhamnose group. **RP4-2** is characterized by a twist in the position α of figure S8.

The same procedure has been adopted for **RP4-2**: the principal conformers found in vacuo are reported in Table S4, the adjusted structure fitting the STM image is compared with the most populated conformer in Figure S10, bottom part, finally the superposition of the structure to the STM image is given in Figure S11 right panel.

Table S4. Results from conformational search in vacuo starting from a polyene chain with a Z kink in α position (Figure S9). Structures have been optimized with no restrain, except for the polyene chain, at M06/tzvp level. Angles reported in the table are defined in figure S6.

	Energy Kcal/mol	Stat. Weight	1	2	3	4	5	6	7
1	0.00	*90.0%*	-101	-59	156	-65	-57	177	-158
2	1.33	*9.4%*	111	-65	154	-67	-60	177	-158
3	3.83	*0.1%*	121	63	158	-64	-59	177	-159
4	4.06	*0.1%*	-118	-173	158	-64	-58	177	-158
5	4.49	*0.0%*	-108	58	127	-70	-59	177	-158
6	4.56	*0.0%*	109	-179	154	-66	-59	177	-159
7	4.60	*0.0%*	107	-179	155	-66	-59	177	-158
8	4.60	*0.0%*	107	-179	155	-66	-59	177	-158
9	4.60	*0.0%*	-112	-66	107	-99	-59	177	-158
10	4.68	*0.0%*	101	180	84	-78	-60	177	-158
11	4.94	*0.0%*	125	-47	-63	-84	-58	177	-159
12	5.49	*0.0%*	121	63	160	-63	-59	172	-70
13	5.93	*0.0%*	-116	-52	-67	-81	-58	177	-158
14	6.09	*0.0%*	103	-180	90	-73	-65	-172	-79
15	6.22	*0.0%*	-112	-67	107	-97	-63	177	-158
16	6.30	*0.0%*	-109	-65	106	-100	-59	172	-70
17	6.62	*0.0%*	100	-64	102	-149	-60	177	-159
18	7.58	*0.0%*	-117	-52	-67	-81	-58	172	-70
19	7.90	*0.0%*	-109	-66	105	-98	-63	172	-70
20	8.24	*0.0%*	-106	66	-175	65	179	177	-159
21	8.25	*0.0%*	102	179	84	-78	-60	-174	56
22	8.89	*0.0%*	104	-180	90	-73	-65	-180	-155
23	11.27	*0.0%*	123	-86	50	-173	-67	-180	-155

5.2 Quantum mechanics/molecular mechanics calculations

RP4 structures on Au(111) have been calculated using a QM/MM approach[S9] with the molecules described with Fireball DFT[S10] and the surface described with interface forcefield.[S11] Minimum energy structure were calculated different initial geometries where we perform at 20 ps Molecular Dynamics at 100 K followed by a final relaxation. The structures chosen were the ones with lowest potential energy for both isomers after the geometrical relaxation. STM simulations were performed using the coordinates of the molecule and gold surfaces where we performed a full Fireball DFT calculation of the electronic structure. This electronic structure is used in combination with PPSTM code[S12] to simulate constant current STM images. Fireball DFT calculations were performed using BLYP exchange-correlation functional with D3 corrections[S13] with norm-conserving pseudo-potentials and a basis set of optimized numerical atomic-like orbitals, s for H, sp^3 for C, N, O and sp^3d^5 for Au.

6. References

(S1) Armistead, B.; Herrero-Foncubierta, P.; Coleman, M.; Quach, P.; Whidbey, C.; Justicia, J.; Tapia, R.; Casares, R.; Millán, A.; Haidour, A.; Granger, J. R.; Vornhagen, J.; Santana-Ufret, V.; Merillat, S.; Adams Waldorf, K., Cuerva, J. M.; Rajagopal, L. Lipid Analogs Reveal Features Critical for Hemolysis and Diminish Granadaene Mediated Group B Streptococcus Infection. *Nat. Commun.* **2020**, *11*, 1502.

(S2) Horcas, I.; Fernández, R.; Gómez-Rodríguez, J. M.; Colchero, J.; Gómez-Herrero, J.; Baro, A. M. WSXM: A Software for Scanning Probe Microscopy and a Tool for Nanotechnology. *Rev. Sci. Instrum.* **2007**, *78*, 013705.

(S3) Caivano, I.; Tošner, Z.; Císařová, I.; Nečas, D.; Kotora, M. A General Synthetic Approach and Photophysical Properties of Regioselectively Fluorinated [5]- and [6]-Helical Bispiroindenofluorenes. *ChemPlusChem* **2020**, *85*, 2010–2016.

(S4) Pracht, P.; Bohle, F.; Grimme, S. Automated Exploration of the Low-Energy Chemical Space with Fast Quantum Chemical Methods. *Phys. Chem. Chem. Phys.* **2020**, *22*, 7169–7192.

(S5) Gaussian 16, Revision C.01, Frisch, M. J.; Trucks, G. W.; Schlegel, H. B.; Scuseria, G. E.; Robb, M. A.; Cheeseman, J. R.; Scalmani, G.; Barone, V.; Petersson, G. A.; Nakatsuji, H.; Li, X.; Caricato, M.; Marenich, A. V.; Bloino, J.; Janesko, B. G.; Gomperts, R.; Mennucci, B.; Hratchian, H. P.; Ortiz, J. V.; Izmaylov, A. F.; Sonnenberg, J. L.; Williams-Young, D.; Ding, F.; Lipparini, F.; Egidi, F.; Goings, J.; Peng, B.; Petrone, A.; Henderson, T.; Ranasinghe, D.; Zakrzewski, V. G.; Gao, J.; Rega, N.; Zheng, G.; Liang, W.; Hada, M.; Ehara, M.; Toyota, K.; Fukuda, R.; Hasegawa, J.; Ishida, M.; Nakajima, T.; Honda, Y.; Kitao, O.; Nakai, H.; Vreven, T.; Throssell, K.; Montgomery, J. A., Jr.; Peralta, J. E.; Ogliaro, F.; Bearpark, M. J.; Heyd, J. J.; Brothers, E. N.; Kudin, K. N.; Staroverov, V. N.; Keith, T. A.; Kobayashi, R.; Normand, J.; Raghavachari, K.; Rendell, A. P.; Burant, J. C.; Iyengar, S. S.; Tomasi, J.; Cossi, M.; Millam, J. M.; Klene, M.; Adamo, C.; Cammi, R.; Ochterski, J. W.; Martin, R. L.; Morokuma, K.; Farkas, O.; Foresman, J. B.; Fox, D. J. Gaussian, Inc., Wallingford CT, 2016.

(S6) Hou, B.; Zhang, T.; Yang, H.; Han, X.; Liu, L.; Li, L.; Grazioli, C.; Wu, X.; Jiang, N.; Wang, Y. Advances in Probing Single Biomolecules: From DNA Bases to Glycans. *Interdiscip Mater.* **2023**, *2*, 511–528.

(S7) Anggara, K.; Zhu, Y.; Delbianco, M.; Rauschenbach, S.; Abb, S.; Seeberger, P. H.; K. Kern, Exploring the Molecular Conformation Space by Soft Molecule–Surface Collision, *J. Am. Chem. Soc.* **2020**, *142*, 21420–21427.

(S8) Anggara, K.; Zhu, Y.; Fittolani, G.; Yu, Y.; Tyrikos-Ergas, T.; Delbianco, M.; Rauschenbach, S.; Abb, S.; Seeberger, P. H.; Kern, K. Identifying the Origin of Local Flexibility in a Carbohydrate Polymer. *Proc. Natl. Acad. Sci. U.S.A.* **2021**, *118*, e2102168118.

(S9) Mendieta-Moreno, J. I.; Walker, R. C.; Lewis, J. P.; Gómez-Puertas, P.; Mendieta, J.; Ortega, J. An Efficient Local-Orbital DFT QM/MM Method for Biomolecular Systems. *J. Chem. Theory Comput.* **2014**, *10*, 2185−2193.

(S10) Lewis, J. P.; Jelinek, P.; Ortega, J.; Demkov, A. A.; Trabada, D. G.; Haycock, B.; Wang, H.; Adams, G.; Tomfohr, J. K.; Abad, E.; Wang, H.; Drabold, D. A. Advances and Applications in the FIREBALL Ab Initio Tight-Binding Molecular-Dynamics Formalism. *Phys. Status Solidi B* **2011**, *248*, 1989−2007.

(S11) Heinz, H.; Lin, T. J.; Kishore Mishra, R.; Emami, F. S. Thermodynamically Consistent Force Fields for the Assembly of Inorganic, Organic, and Biological Nanostructures: the INTERFACE Force Field. *Langmuir* **2013**, *29*, 1754−1765.

(S12) Krejcí̌, O.; Hapala, P.; Ondrácek,ˇ M.; Jelínek, P. Principles and Simulations of High-Resolution STM Imaging with a Flexible Tip Apex. *Phys. Rev. B* **2017**, *95*, 045407.

(S13) Grimme, S.; Ehrlich, S.; Goerigk, L. Effect of the Damping Function in Dispersion Corrected Density Functional Theory. *J. Comput. Chem.* **2011**, *32*, 1456−1465.
